# Breast Cancer and Microcalcifications: An Osteoimmunological Disorder?

**DOI:** 10.3390/ijms21228613

**Published:** 2020-11-15

**Authors:** Alisson Clemenceau, Laetitia Michou, Caroline Diorio, Francine Durocher

**Affiliations:** 1 Department of Molecular Medicine, Faculty of Medicine, Laval University, Quebec, QC G1V 0A6, Canada; alisson.clemenceau@crchudequebec.ulaval.ca; 2 Cancer Research Centre, CHU de Quebec Research Centre, Quebec, QC G1V 4G2, Canada; caroline.diorio@crchudequebec.ulaval.ca; 3 Department of Medicine, Laval University, Quebec, QC G1V 0A6, Canada; laetitia.michou@crchudequebec.ulaval.ca; 4 Department of Rheumatology, CHU de Québec-Université Laval, Quebec, QC G1V 4G2, Canada; 5 CHU de Québec-Université Laval Research Centre, Quebec, QC G1V 4G2, Canada; 6 Department of Preventive and Social Medicine, Faculty of Medicine, Laval University, Quebec, QC G1V 0A6, Canada

**Keywords:** osteoimmunology, osteoimmunological disorders, breast cancer, lobular involution, microcalcifications, hydroxyapatite, inflammation, T-cells, osteoblast-like cells, osteoclast-like

## Abstract

The presence of microcalcifications in the breast microenvironment, combined with the growing evidences of the possible presence of osteoblast-like or osteoclast-like cells in the breast, suggest the existence of active processes of calcification in the breast tissue during a woman’s life. Furthermore, much evidence that osteoimmunological disorders, such as osteoarthritis, rheumatoid arthritis, or periodontitis influence the risk of developing breast cancer in women exists and vice versa. Antiresorptive drugs benefits on breast cancer incidence and progression have been reported in the past decades. More recently, biological agents targeting pro-inflammatory cytokines used against rheumatoid arthritis also demonstrated benefits against breast cancer cell lines proliferation, viability, and migratory abilities, both in vitro and in vivo in xenografted mice. Hence, it is tempting to hypothesize that breast carcinogenesis should be considered as a potential osteoimmunological disorder. In this review, we compare microenvironments and molecular characteristics in the most frequent osteoimmunological disorders with major events occurring in a woman’s breast during her lifetime. We also highlight what the use of bone anabolic drugs, antiresorptive, and biological agents targeting pro-inflammatory cytokines against breast cancer can teach us.

## 1. Introduction

**Figure 1:**
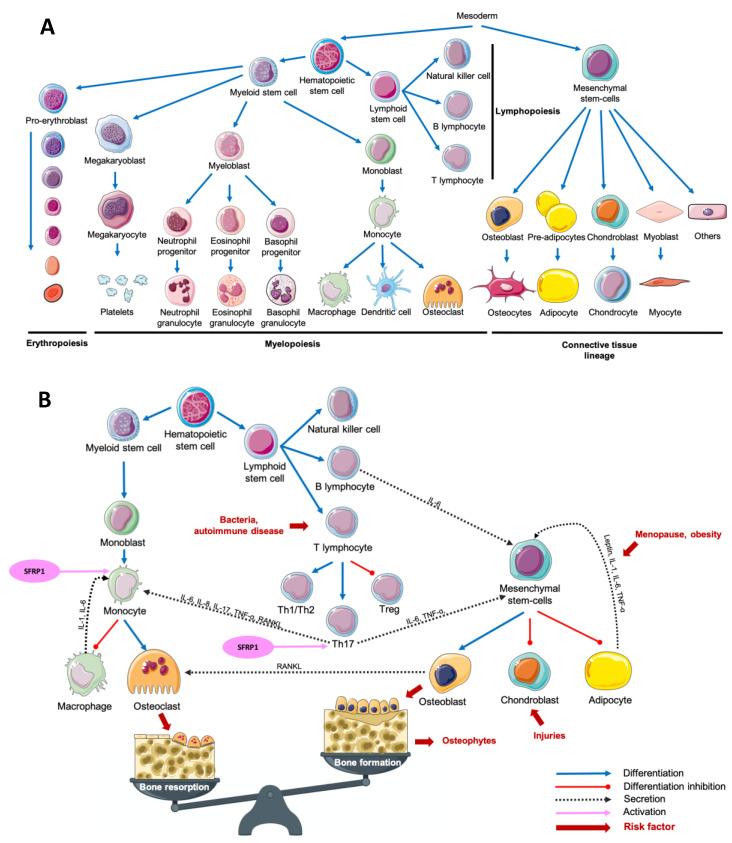
Main cell lineages (**A**) and their crosstalk in an osteoimmunological context (**B**). Abbreviations: IL = interleukin, RANKL = Receptor Activator of Nuclear factor Kappa-B Ligand, SFRP1 = Secreted Frizzled-Related Protein 1, Th = T helper, TNF-α = Tumor Necrosis Factor-alpha, Treg = T regulator.

The term “osteoimmunology” was first used in 2000 by Choi et al. to define a new paradigm describing the crosstalk between the immune system and osteoclastogenesis [1]. The multiplicity of osteoimmunological disorders is due to the variety of stimuli responsible for the immune system activation. In fact, adaptative and innate immunity could be induced by several pathological (bacteria, tissue injury) or physiological (tissue remodeling) processes. Despite this variety of stimuli, the associated response remains similar between diseases: a crosstalk between myeloid lineage, mesenchymal stem cells and the inflammatory microenvironment, which results mainly in both excessive bone and cartilage resorption, and chronic inflammation, but also in bone formation in some cases (Figure 1) [1,2,3,4,5,6].

The female breast is a fascinating organ, which undergoes incredible tissue remodeling throughout a woman’s life. From the mammary gland branching, to the age-related lobular involution through lactation and postlactation lobular involution, breast history is a complex process that we have to understand to better decipher carcinogenesis. Most of the processes previously cited are characterized by a transitory physiological inflammatory microenvironment that takes place in the breast tissue, combined with an immune system recruitment, both influencing epithelial cells proliferation, migration and mesenchymal stem cells differentiation. If the role of colostrum and the impact of milk composition on newborn’s health is extensively studied, the impact of lactation on a mother’s breast and her global health remains unknown, and underexplored. Indeed, if both parity and breastfeeding are associated with a decreased risk of breast cancer development, the biological and molecular causes of such protective effects remain poorly studied [7,8,9,10]. As a natural weapon against breast tumorigenesis, age-related lobular involution has been studied as a risk factor of breast cancer development [11,12,13,14,15,16,17,18,19,20,21,22,23,24]. However, similar to the lactation process, only a few studies have explored its biological process and its potential causal role in early breast tumorigenesis.

Breast microcalcifications are composed of either calcium oxalate crystals, which are associated with benign breast lesions, or hydroxyapatite crystals, associated with both benign and malignant lesions. Microcalcifications appearance at mammography and composition are currently taken into account for breast cancer risk and prognostic stratifications [25,26]. The presence of microcalcifications in the breast microenvironment, combined with the growing evidences of the presence of osteoblast-like and osteoclast-like cells in the breast, suggest the existence of active processes of calcification in breast tissue during a woman’s life [27,28]. However, the crosstalk between microcalcifications and the breast microenvironment throughout a woman’s life, and their implications in early breast carcinogenesis remain misunderstood.

Interestingly, much evidence that osteoimmunological disorders such as osteoarthritis [29], rheumatoid arthritis [30,31,32,33,34] or periodontitis [35,36,37] influence the risk of developing breast cancer in women exists and vice versa. Furthermore, antiresorptive drugs benefits on breast cancer incidence and progression in women has been reported in the past decades [38,39,40,41]. Anti-cytokines drugs used against rheumatoid arthritis also demonstrated benefits against breast cancer cell lines proliferation, viability and migratory abilities both in vitro and in vivo in xenografted mice [42,43,44,45,46,47,48]. Hence, it is tempting to hypothesize that breast carcinogenesis associated with microcalcifications should be considered a potential osteoimmunological disorder. In this review, we compare microenvironments and molecular characteristics in the most frequent known osteoimmunological disorders with major events occurring in a woman’s breast during her lifetime with an emphasis on breast cancer and the risk of breast cancer. In addition, we highlight what the use of bone anabolic drugs, antiresorptive and biological agents targeting on pro-inflammatory cytokines against breast cancer can teach us.

## 2. Overview of the Most Frequent Osteoimmunological Disorders

### 2.1. Osteoporosis (OP)

Osteoporosis (OP) is a metabolic disease characterized by a loss of bone mass and an excessive fragility of bones due to an imbalance between bone resorption and bone formation. This multifactorial disease is notably due to an increased secretion of pro-inflammatory cytokines and adipokines, inducing an excessive osteoclastogenesis. Estrogen deprivation after menopause is an important OP risk factor. Estradiol serum level is inversely proportional to the risk of fractures [49,50,51]. Postmenopausal OP was first associated with an excessive inflammatory reaction following the decrease of estrogens production in 1991. After only 2 weeks following oophorectomy, the authors have observed an increased urinary concentration of Interleukin-1 (IL-1) and Tumor Necrosis Factor-Alpha (TNF-α) compared with premenopausal women [52]. Interestingly, it was reported than an increased production of TNF-α and Receptor Activator of Nuclear factor Kappa-B Ligand (RANKL) in postmenopausal OP women was associated with an overactivation of T-cells responsible for an increased osteoclasts formation (Figure 1B) [53,54,55]. Leptin, an adipokine well known to be involved in food intake and energy metabolism, is also associated with bone metabolism. By fixing leptin receptor (Ob-R) on mesenchymal stem cells (MSCs), leptin stops adipocytic differentiation while it enhances osteoblastic differentiation and proliferation (Figure 1B) [56,57]. Surprisingly, the adipogenic differentiation of MSCs is higher in MSCs from postmenopausal women with OP compared with postmenopausal women without OP. While leptin is responsible for an antiadipogenic differentiation in postmenopausal women without OP, it had no effect on MSCs obtained from postmenopausal women with OP [58]. Interestingly, it is not only the secretion of cytokines and adipokines that seems to be dysregulated in OP. In bone microenvironment, Secreted Frizzled-Related Protein 1 (SFRP1), a Wnt canonical and non-canonical signaling pathway antagonist [59,60,61,62,63], is known to regulate the differentiation, proliferation and apoptosis of osteoblasts and osteocytes [64,65]. In fact, SFRP1 promotes MSCs differentiation in adipocytes and preadipocyte maturation, decreasing osteoblastogenesis (Figure 1B) [66,67,68]. Furthermore, by regulating the osteoblasts-induced osteoclastogenesis, SFRP1 is also involved in bone resorption process [69]. Interestingly, Tang et al. observed that SFRP1 and miR-144 serum levels were higher and positively correlated in postmenopausal osteoporotic women compared with postmenopausal women with normal bone density. They also reported that miR-144 promotes osteoblastic differentiation of bone marrow-derived MSCs by targeting SFRP1 [70]. SFRP1 was also reported as down-regulated in the bone marrow of OP patients by Gu et al. [71]. In summary, we observe both a decrease of leptin-induced osteoblasts differentiation and an increased osteoblasts-induced osteoclastogenesis modulated by SFRP1. This suggests that the adipose tissue has a crucial role in bone metabolism and its dysregulation can promote metabolic disorders like OP. As reviewed by Kothari et al., adipose tissue is also a crucial player in breast tissue remodeling and carcinogenesis [72]. The relationship between osteoporosis and breast cancer is puzzling. Both diseases affect principally postmenopausal women after 65 years old. However, the biological explanation of such link remains misunderstood. OP risk decreases with estrogen exposure while breast cancer risk increases. Consequently, an older age at menarche and a younger age at menopause increase OP risk while they decrease breast cancer risk. On the other hand, weight under 55 kg at menopause increases OP risk, while obesity increases breast cancer risk in postmenopausal women [73]. A comparative study of both common and opposite biological and molecular aspects of both diseases could help to better manage women health.

### 2.2. Osteoarthritis

Osteoarthritis (OA) is a degenerative disorder of the joints induced by an increasing catabolic activity in both cartilage and bone tissues. OA is also described as a chronic wound due to initial cartilage injuries, inducing pro-inflammatory cytokines secretion in the synovial fluid and the associated immune system recruitment to repair injuries. Among the pro-inflammatory markers up-regulated in OA context, leptin, TNF-α, interleukin 6 (IL-6) and IL-1, known to negatively regulate cartilage formation [56,57,58,74,75]. IL-1 and TNF-α are produced by activated chondrocytes and synoviocytes in early OA and by leukocytes such as macrophages, T-cells and B-cells, which will then secrete many other pro-inflammatory cytokines including IL-6. Activated T helper 17 cells (Th17) increase interleukin 17 (IL-17) level in synovial fluid which is associated with an increased RANKL level, resulting in higher osteoclastogenesis (Figure 1B) [76,77]. It was reported that leptin controls not only bone formation but also bone resorption. By modulating RANKL expression, leptin decreases osteoclastic differentiation through the Beta-2 adrenergic receptor (ADRB2) expressed by osteoblasts [78]. SFRP1, which is also involved in osteoclastogenesis regulation is secreted by synovial cells and predominantly by fibroblasts-like cells of the synovial fluid in an OA context. However, Pasold et al. observed that in OA mouse models, SFRP1 expression is reduced in chondrocytes and MSCs, resulting in preferential osteoblastogenesis compared with chondrogenesis [79]. This increased osteoblastogenesis results in osteophytes production while the decreased chondrogenesis prevents cartilage healing resulting in a chronical inflammatory disorder. Once more, a subtle imbalance due to cartilage injuries in the joints results in MSCs preferential differentiation toward osteoblastic lineage compared with chondrocyte differentiation, which is needed to achieve cartilage healing. Interestingly, patients with knee or hip arthritis have a higher risk of breast cancer development after adjustment for age and sex [29]. However, adjustment for mammary gland history or stratification for histopathological characteristics of breast cancer lesions was unavailable. To date, references are insufficient to clearly understand the impact of OA on breast cancer risk.

### 2.3. Rheumatoid Arthritis

In contrast with OA which is initiated by cartilage lesions, rheumatoid arthritis (RA) is an autoimmune disease characterized by an uncontrolled immune reaction against both cartilage and bone tissue. RA development is associated with genetic predispositions and the presence of T-cell receptors at the joints [80,81]. Consequently, the recruitment of T-cells, notably T helper 1 (Th1) and Th17, results in IL-17 and TNF-α production, responsible for the increased production of IL-1 and IL-6 by macrophages and dendritic cells. This microenvironment promotes Th17 differentiation to the detriment of T regulators (Treg) differentiation. To complete the loop, IL-1, IL-6, IL-17, and TNF-α are known to stimulate osteoclastogenesis, which results in the degradation of mineralized tissue such as mineralized cartilage and subchondral bone [81,82,83]. Similar to what is observed in OA, leptin serum levels are higher in RA patients, so that overweight and obesity have been associated with RA [84,85]. MSCs were found in the synovium in a RA context, and, as described before, their differentiation is finely regulated by the microenvironment composition [86,87]. The administration of anti-Dikkopf-1 (Dkk-1) antibody in RA mouse models induces a decrease of bone erosion, potentially due to a decrease of osteoclast differentiation in the joint by decreasing levels of RANKL [88]. Lee et al. observed that the addition of SFRP1, another Wnt signaling antagonist in naïve T-cells medium is responsible for Th17 polarized T-cells differentiation. They also demonstrated that this differentiation is due to an increased sensitivity of T-cells to Transforming Growth Factor-Beta (TGF-ß) [89]. In murine models of arthritis, Matzelle et al. observed that the resolution of the inflammation resulted in a down-regulation of SFRP1 expression, a Wnt signaling antagonist. Consequently, by activating the Wnt signaling pathway, they also observed a decrease of bone resorption combined with an induction of osteoblast mineralization [30]. In breast, SFRP1 expression is higher during age-related lobular involution and in presence of microcalcifications compared with patients completely involuted and without microcalcification, respectively [90]. Interestingly, the incidence of RA in breast cancer patients is lower compared with patients without breast cancer after adjustment for age, comorbidities and breast cancer treatments [31]. However, the incidence of breast cancer in RA patients remains controversial. Bhandari et al. observed a higher cancer prevalence in RA patients, with a high proportion of breast cancer [32]. On the other hand, the meta-analysis of Tian et al. showed that the breast cancer risk in RA patient was not increased versus in the general population. However, when the population study was stratified for ethnicity, RA patients breast cancer risk was increased in non-Caucasian population while it decreased in the Caucasian population [33]. More recently, Wadström et al. observed a decreased occurrence of breast cancer in RA patients, also observable after adjustment for breast cancer treatment, suggesting that this reduction of breast cancer risk was already present before breast cancer treatment administration [34]. Unfortunately, the studied cohorts were not stratified for the presence of microcalcifications, the parity history or the degree of lobular involution, which could be a potential way of investigation to better understand the link between both diseases.

### 2.4. Periodontitis

This multibacterial-induced inflammatory disease is characterized by the destruction of periodontal tissues, a loss of alveolar bone mass principally due to an exacerbation of osteoclastogenesis, an inflammatory cells infiltration and an increased fibroblasts apoptosis. More precisely, after antigenic activation of T-cell surface glycoprotein CD4 positive (CD4+) naïve T-cells, activated Th1, T helper 2 (Th2), and Th17 produce cytokines responsible for the activation of B-cells, dendritic cells and neutrophils. Then, activated B-cells and T-cells produce RANKL responsible for an increased osteoclastogenesis [91,92,93,94,95,96]. Interestingly, Kawai et al. demonstrated that in healthy gingival tissue, only 20% of B-cells ant T-cells expressed RANKL. On the other hand, 50% of T-cells and 90% of B-cells expressed RANKL in a periodontitis (PD) context, which results in an abnormal alveolar bone destruction [93]. The clonal activation of B-cells induces the production of antibodies against gingival components such as collagen, resulting in the destruction of periodontal tissue. Numerous pro-inflammatory cytokines are upregulated in a periodontitis context including IL-1, IL-6, Interleukin-8 (IL-8), and TNF-α. The lack of Treg to control this inflammation completes the loop, and chronical inflammation then takes place in the periodontal tissue [91,92,93,94,95,96]. Leptin was also reported as upregulated in human saliva and circulating blood [97], and in dog periodontal ligament tissue [98] in a PD context. More recently, Zhu et al. performed a meta-analysis highlighting elevated leptin serum level and lower adiponectin serum level in PD patients compared with controls in the group with a body mass index under 30 [99]. Li and Amar reported that anti-SFRP1 antibody was able to reverse both osteoclastogenesis and related inflammation, suggesting its crucial role in bone remodeling processes [100]. Surprisingly, multiple studies reported that PD is associated with breast cancer development, suggesting that this disorder could be a risk factor of breast cancer development [35,36,37]. However, no observation regarding a potential causal role of PD-related molecular issues on breast cancer development was reported yet. Investigations are still needed to conclude the existence of a link between PD and breast cancer development.

### 2.5. Other Osteoimmunological Disorders

The risk of developing an autoimmune rheumatic disease such as RA, systemic lupus erythematosus (SLE) or systemic sclerosis (SSc) in patients with breast cancer is lower compared with age and year of index date matched patients without breast cancer [31]. Reciprocally, the risk of developing breast cancer in SLE patients is lower compared with the general population [101,102,103,104,105,106]. On the other hand, Colaci et al. observed a higher incidence of breast cancer in SSc patients compared with age-sex-matched patients without SSc [107]. The same scheme is observable in psoriatic arthritis (PsA) patients. While some groups reported that breast cancer incidence was higher in PsA patients compared with age-sex-matched patients without PsA [108], others observed no difference in breast cancer occurrence between the two groups [109]. However, obesity and overweight incidence and prevalence in PsA patients are higher compared with the general population [110,111,112]. Obesity is a risk factor of triple-negative breast cancer development particularly in premenopausal women [113,114] and it is associated with poor breast cancer survival [115]. The higher breast cancer rate in the PsA group could be explained by a higher proportion of obese patients in the PsA group. Unfortunately, Wilton et al. did not report the body mass index (BMI) nor the breast cancer molecular subtype of the population studied. Divergent results could also potentially be explained by the absence of the cohort stratification regarding the degree of lobular involution, and the presence of microcalcifications. As described above, multiple studies reported a change in breast cancer prevalence or incidence in patients with osteoimmunological disorders, as well as few evidences of breast cancer effects on osteoimmunological disorders occurrence independently of breast cancer treatment. However, many adjustments for clinical variables as well as stratified analyses are lacking. Consequently, confounding variables are potentially responsible for multiple controversial results. Extensive studies are needed to conclude to the existence a potential link between osteoimmunological disorders and breast cancer incidence and this represent a new avenue of investigation to better personalize breast cancer treatment.

## 3. Osteoimmunology of the Breast Tissue during a Woman’s Lifetime

### 3.1. Pregnancy and Lactation

During lactation, a Ca^2+^ related gene expression program named CALTRANS takes place, including Ca^2+^-ATPases, pumps or channels to enrich milk with Ca^2+^ [116]. Interestingly, *Plasma Membrane Ca^2+^-ATPase* (*PMCA2*) dysregulation is associated with microcalcifications, breast cancer development and poor prognosis [117]. Case-reports of pseudo-lactational hyperplasia were previously described in both premenopausal and postmenopausal women. These women sustained pregnancy-like changes, including mammary gland branching and microcalcifications development independently from pregnancy and lactation [118]. This was also observed in virgin mice deficient for *Sfrp1* expression, exhibiting mammary glands branching and lobuloalveolar activity similar to mid-pregnant wild type mice [119]. In cows, during the late peak of lactation, SFRP1 is significantly upregulated suggesting its involvement in the acute inflammatory phase needed to initiate lobular involution [120]. In mice, leptin expression is upregulated before lactation while it is downregulated at mid-lactating stage. However, the leptin serum concentration was the same at both stages, suggesting a regional production of the mammary gland and associated adipose tissue [121]. The leptin fixes Ob-Rb, which, after dimerization, will activate both tyrosine-protein kinase JAK (JAK)/signal transducer and activator of transcription (STAT) and mitogen-activated protein kinase (MAPK)/ extracellular signal-regulated kinase (ERK) signaling pathways. Interestingly, the abrupt end of lactation in mice results in an increased inflammation of the mammary gland tissue, an hyperplasia and an exacerbation of estrogen receptor-alpha (ER-α) expression [122]. This could be explained by the fact that leptin production by epithelial cells and adipocytes decreases gradually during lactation [121]. Leptin receptor Ob-Rb colocalizes with ER-α on rats hypothalamic neurons, suggesting a crosstalk between leptin and estrogens peripheral signaling [123,124]. These evidences suggest the importance of a successful postlactation lobular involution to avoid microcalcifications and to prevent breast cancer development. It also suggests the existence of microcalcifications formation by both tumoral and non-tumoral breast epithelial cells due to CALTRANS dysregulations, in parous and nulliparous women. Breast microcalcifications formation, independently of the presence of osteoblast-like cells suggests the existence of multiple microcalcifications formation processes, which are crucial to better characterized to better characterize and prevent breast cancer development.

### 3.2. Postlactation Lobular Involution

Postlactation involution is initiated by an acute inflammatory phase due to the accumulation of milk in the alveolar lumen in response to the end of suckling. This first reversible step induces the decrease of milk production and the apoptosis of the epithelial cells into the acinar lumen [122,125]. Among the cytokines produced during the acute phase, IL-1 and TNF-α are involved in the nuclear translocation of Nuclear Factor-Kappa B (NF-κB) and IL-6 is responsible for Signal Transducer and Activator of Transcription 3 (STAT3) activation [126]. The localized process of lobular involution initiation is followed by a massive systemic fall of hormones which results in the second part of lobular involution. STAT3 is involved in both the acute phase and the lobular involution, and notably by regulating apoptosis [125,126,127,128]. This acute inflammatory phase is needed for early recruitment of dendritic cells, followed by macrophages and T-cells, consistent with the wound healing program [129]. In addition, the transdifferentiation of pink adipocytes, present only during pregnancy and lactation, in white adipose tissue remains essential for proper involution. Interestingly, this process is mediated by Osteopontin (*OPN*), which is highly expressed during lactation and involution while it is not found in other stages of breast development [72,130,131]. *OPN*, also named Secreted Phosphoprotein 1 (SPP1) is also expressed by osteoblasts and is an agonist of bone resorption processes. *OPN* is overexpressed in breast cancer tissue in the presence of hydroxyapatite crystals compared with non-calcified tissue, but also in tumoral calcified tissue compared with non-tumoral calcified tissue suggesting its involvement in both calcification and tumorigenesis [132]. In addition, mRNA *OPN* expression is also associated with tumor aggressiveness and invasiveness [133,134]. By silencing *OPN* in MDA-MB-231 triple-negative breast cancer cell line (estrogen receptor (ER), progesterone receptor (PR) and receptor tyrosine-protein kinase erbB-2 (HER2) negative), a decrease in the production of hydroxyapatite crystals in an osteogenic medium and a decrease of cell migration were observed [135]. An excess of involution-related adipogenesis in the breast microenvironment is associated with an excessive production of adipokines [72]. To fully measure the importance of lobular involution characterization, McDaniel et al. isolated mammary glands extracellular matrix from nulliparous rats, and rats undergoing postlactation involution. Tissues from nulliparous animals promoted the ductal organization of the MCF-12A non-tumoral cell line and stopped the invasion of the MDA-MB-231 triple negative breast cancer cell line. Inversely, mammary gland tissue from rats undergoing involution did not promote ductal organization of MCF-12A while it promoted MDA-MB-231 invasiveness [136]. This suggests a fragile balance between the effects of lobular involution-related inflammation on non-tumoral tissue versus tumoral tissue that remains urgent to understand, especially to improve lactating breast cancer treatment.

### 3.3. Age-Related Lobular Involution

Age-related lobular involution is a perimenopausal process starting progressively around 40 years old and aims to replace epithelial cells and stroma by adipose tissue and collagen to decrease the risks of breast hyperplasia and tumor development. To do so, the breast undergoes a significant tissue remodeling, requiring inflammation, apoptosis, immune system recruitment, extracellular matrix destruction and adipogenesis. Hanna et al. described the inflammatory profile associated with age-related lobular involution and highlighted that chronic inflammation could reduce the age-related involution completion and consequently, increase breast cancer risk. A higher expression of the pro-inflammatory markers such as IL-6, TNF-α, C-reactive protein (CRP), cyclooxygenase 2 (COX-2), leptin, serum amyloid 1 (SAA1) and IL-8 were inversely associated with completed lobular involution while a higher expression of the anti-inflammatory marker interleukin 10 (IL-10) [137] was positively associated with mammographic density, another well-known breast cancer risk factor [14]. Interestingly, IL-6, TNF-α and leptin expression by epithelial breast tissue is strongly correlated with SFRP1 expression, also known to be inversely associated with completed age-related lobular involution. SFRP1 is a good predictor of the degree of lobular involution and its expression is higher in presence of microcalcifications. Moreover, SFRP1 expression is lower in involuted nulliparous women compared with involuted non-nulliparous women suggesting that the first lobular involution is different from the others [90]. This is particularly important to consider as lower expression of SFRP1 in breast tissue is largely associated with breast cancer development and poor prognosis [134,138,139,140,141,142,143,144,145,146]. Leptin serum level is higher following estrogen deprivation-related adipogenesis in ovariectomized rats compared with controls [147]. The impact of concomitant leptin expression by breast adipose tissue during lobular involution and a higher leptin serum level due to menopause-related fall of estrogen remains unexplored. However, leptin is involved in preferential osteoblastic differentiation of MSCs. Naseem et al. observed that the proportion of patients with microcalcifications was higher in perimenopausal women compared with both premenopausal and postmenopausal women [148]. This result suggests the existence of a potential microcalcification resorption process in breast tissue and proves the importance to decipher the molecular mechanisms of lobular involution process to better prevent breast cancer development and recurrence in women diagnosed for breast cancer before 40 years old.

### 3.4. Inflammation in Breast Carcinogenesis

**Figure 2:**
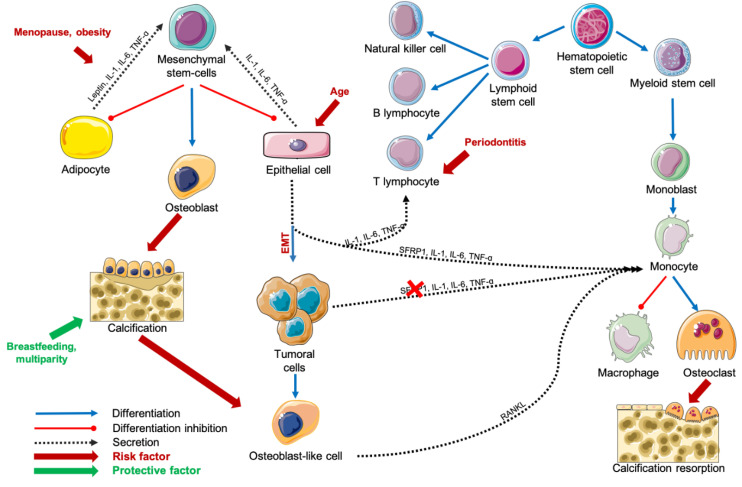
Schematization of carcinogenesis during age-related lobular involution process. Abbreviations: IL = interleukin, RANKL = Receptor Activator of Nuclear factor Kappa-B Ligand, SFRP1 = Secreted Frizzled-Related Protein 1, TNF-α = Tumor Necrosis Factor-alpha.

As inflammation is needed in several physiological processes of mammary gland involution, it remains difficult to segregate which, from the “good” or the “bad” inflammations could exacerbate breast cancer risk (Figure 2). Breast cancer is nowadays, the leading cancer type diagnosed in women and the second leading cause of cancer related death in women. One in eight women will develop an invasive breast cancer during her life. The 5-year survival rate for all stages of breast cancer in 2020 is 90%. However, the 5-year survival rate in distant disease patients is 27% only [149]. These statistics testify that both breast cancer prevention and screening are essential to avoid late diagnosis. However, knowledge regarding carcinogenesis-related molecular pathways is still lacking. Chronic inflammation is associated with an increasing risk of breast cancer [150]. In non-tumoral breast tissue, higher expression of IL-6 is associated with higher mammographic density, while higher expression of anti-inflammatory TGF-ß is associated with lower mammographic density in both premenopausal and postmenopausal women [151]. Furthermore, as described previously, local inflammation is inversely associated with age-related lobular involution [137]. Similar to osteoimmunological disorders cited above, breast cancer could also be promoted by the immune system. At first, tumor-infiltrating lymphocytes, composed by a majority of T-cells and macrophages perform immunosurveillance and engage the destruction of malignant cells. However, in a second phase, they promote both proliferation and escape of immune system resistant tumoral cells [152]. Confirming that evidence, Desmedt et al. reported that tumoral-infiltrating cells were preferentially detected in in situ lesions compared with invasive lesions [153]. In vitro, invasive ductal carcinoma (IDC) supernatants contained more inflammatory cytokines and less anti-inflammatory cytokines compared with non-invasive breast lesions supernatant, suggesting that an inflammatory profile is associated not only with cancer risk, but also with cancer aggressivity [154]. Interestingly, 90% of ductal carcinoma in situ (DCIS) contain microcalcifications, and 40% become invasive. Microcalcifications are currently diagnosed by mammography and used to perform malignancy risk stratification of breast lesions [26]. However, the impact of microcalcifications in breast cells microenvironment at the molecular level as well as the interactions between such crystals and breast cells remains underexplored. Thought, Naseem et al. observed that microcalcifications are associated with HER2 overexpression, invasiveness, perimenopausal status, heterogeneous breast density and multifocal disease [148]. These evidences suggest that inflammatory profile of the breast tissue during a woman lifetime could have a major impact on microcalcifications development and carcinogenesis. Consequently, it appears essential to consider microcalcifications as a potential active player in breast cancer development.

## 4. Evidence of the Presence of Osteoblastic and Osteoclastic Lineages in the Breast Tissue

### 4.1. Osteoblast-Like Breast Cells

Multipotent stem cells are necessary to ensure tissue renewal as well as injury repair in adults. More precisely, multipotent stem cells are immatured cells with an unlimited capacity of self-renewal. Furthermore, these cells can differentiate into all cell types of few restricted lineages. Both embryonic and adult breast tissue developments are regulated by the Wnt signaling pathway and notably by SFRP1 [155,156,157,158]. However, we reported throughout this review that SFRP1 is dramatically modulated during breast history. It is now well described that not only stem cells have multipotency abilities in adult tissues. In fact, epithelial to mesenchymal transition (EMT) results in the acquisition of multipotency and invasiveness capacities largely associated with cancer development. In 2016, Tan et al. reported that MCF-7 and T47D breast cancer cell lines cultured in conditioned medium from cancer-associated fibroblasts underwent EMT [159]. Scimeca et al. reported that human breast cancer lesions exhibited mesenchymal markers that correlated with the quantity of breast-osteoblast-like cells [160]. The quantity of breast-osteoblast-like cells was positively correlated with TGF-ß and vimentin expression while it was inversely correlated with CD44 expression [160]. Interestingly, they also reported that the expression of CD44 and Vimentin was higher in presence of microcalcifications compared with tumor tissue without microcalcifications [161]. Tulotta et al. reported that the contact between breast cancer cells and osteoblasts or bone marrow cells was responsible for the increased secretion of IL-1ß by the three protagonists, resulting in EMT and tumor aggressiveness [44]. In 1985, Valentin-Opran et al. reported for the first time an estrogen-dependent bone resorbing activity from the MCF-7 luminal A (ER and/or PR positive, and HER2 negative) breast cancer cell line in vitro [162,163]. At that time, this result was used to explain acute hypercalcemia associated with ER-positive tumors and osteolytic characteristics of bone metastases derived from breast cancer lesions but the local effect of an osteoblast stimulating activity was not explored. In 2012, Cox et al. investigated the origin of breast microcalcifications and reported that hydroxyapatite microcalcifications were actively produced by breast cancer cells with osteoblastic characteristics in vitro [164,165]. In 2016, Tan et al. reported that breast cancer tissues expressed more bone-related genes compared with non-tumoral-tissues. Among these genes, *Runt-related transcription factor 2* (*RUNX2*) is a key regulator of bone-related gene expression in breast cancer cell lines [159]. More recently, Scimeca et al. reported that the expression of osteoblasts specific proteins such as RANKL, OPN, vitamin D3 receptor (VDR), and bone morphogenetic protein 2 (BMP-2) was higher in breast cancer lesions with microcalcifications compared with breast cancer lesions without microcalcifications [161]. To complete the investigation, they also quantified mineralization process markers like bone morphogenetic protein 4 (BMP-4) and pentraxin-related protein PTX3 (PTX3), and their expression was also higher in presence of microcalcification compared with breast cancer lesions without microcalcifications [161]. More and more evidences of osteoblast-like cell existence, as well as breast cancer cell line abilities to produce calcifications, are reported. However, their impacts on breast non-tumoral and tumoral cells remain poorly understood and their origins are still unknown.

### 4.2. Osteoclast-Like Giant Breast Cells

For the first time in 1979, breast osteoclast-like giant cells were described by Agnantis et al. who studied eight infiltrating carcinomas containing these cells of interest [166]. This rare cell type is defined as a giant multinucleated cell similar to histiocytic osteoclasts found in bone tissue. However, if osteoclast-like giant cells are similar to osteoclasts regarding the abilities of resorbing cortical bone, they do not have ruffled border or clear zone. In addition, unlike osteoclasts which are coupled with osteoblasts, PTH alone is able to activate bone resorption by osteoclast-like giant cells in vitro [167]. Interestingly, cases reported were aged 43 to 84 with a median age of 49 years old, suggesting that this type of lesion is not menopausal-status specific [166]. Since then, around 200 breast cancer cases with osteoclast-like giant cells in both in situ and infiltrating lesions were reported [168,169,170,171,172,173,174,175,176]. Breast osteoclast-like giant cells were positive for CD68 [171,172,175,177] and CD163 [174] staining confirming their monocytic lineage. Tumoral-cells were ER and PR positive [168,171,172,174], HER2 negative [168,171,172], and associated stromal cells were positive for vimentin expression when tested [171]. Few triple-negative breast carcinomas with osteoclastic giant cells were also reported [169,177,178]. Interestingly, breast osteoclast-like giant cells associated with tumoral tissue and invasiveness are predominantly CD163 positive, hence possessing a M2-macrophage phenotype [178]. Cancer cells treatment with leptin induces the secretion of both intracellular adhesion molecule 1 (ICAM-1) and RANKL and enhances tumor-induced osteolysis in vivo, suggesting a potential role for leptin in osteoclast-like giant cell formation in a cancer context [179]. However, the scarcity of available references and reported cases, combined with the major differences between the phenotypic characteristics of these multinucleated giant cells compared with osteoclasts show that we must remain cautious regarding any conclusions.

## 5. Osteoblastic and Osteoclastic Breast Cancer Metastases in Bone Microenvironment

### 5.1. Osteolytic Lesions

In bone microenvironment, breast cancer metastases form in 80 to 90% of osteolytic lesions which supports an excessive bone resorption and degradation. Osteolytic lesions express bone resorption promoting factors, such as parathyroid hormone-related protein (PTHrP), which is higher in both bone metastases and serum of osteolytic bone metastases from patients [180,181]. However, PTHrP increases RANKL expression by osteoblasts and decreases osteoprotegerin (OPG) expression, resulting in osteoclast activation [182]. Furthermore, Bendre et al. observed in vitro that IL-8 expression by breast cancer cells could promote osteoclastogenesis indirectly by promoting RANKL expression by osteoblasts, and directly by inducing blood mononuclear cells differentiation in resorbing osteoclasts [183]. Furthermore, neutralizing IL-1ß with anakinra in mice xenografted with triple negative MDA-MB-231 and luminal A MCF-7 breast cancer cell lines reduces the incidence of bone metastases and bone turnover markers expression including TNF-α [42]. Kovacheva et al. observed that knockdown MDA-MB-231 for OPN induces a decrease of cell proliferation and migration abilities, and a remission of the osteolytic lesions compared with unmodified MDA-MB-231 [184]. Dkk-1, a Wnt signaling antagonist, is expressed in osteolytic lesions while it was not expressed in osteoblastic lesions [185]. Furthermore, Dkk-1 level in the serum of women with breast cancer bone metastases is higher compared with age-matched healthy controls and patients with breast cancer metastases in another site than bones [186]. Interestingly, ER negative breast cancer cell lines MDA-MB-231, MDA-MB-435s and BT549 form osteolytic lesions [187]. However, the proportions of osteolytic lesions by breast cancer molecular subtypes were not reported in women yet. Furthermore, the question about which, of metastases secretions, breast cancer cells with osteoblastic phenotype or both are responsible for bone resorption remains without an answer so far.

### 5.2. Osteoblastic and Mixed Lesions

Osteoblastic breast cancer bone metastases occur in 10 to 20% of breast cancer bone metastases and show disorganized new bone with an associated increase of bone resorption. These lesions support osteoblastogenesis notably by expressing TGF-ß, bone morphogenic proteins, and Wnt proteins, all known to promote osteoblasts formation [188]. Another potential causal factor of breast cancer cell mediated osteoblastic lesions is Endothelin-1 (*ET-1*). *ET-1* is higher in male serum with prostate cancer compared with male without prostate cancer. Furthermore, prostate cancer cell lines expressing *ET-1* increase phosphatase alkaline activity in new bone formation [189]. Interestingly, metastases obtained from luminal A breast cancer cell lines MCF-7, ZR75.1 and T47D also form osteoblastic lesions [187,190]. Yin et al. demonstrated that a treatment against *ET-1* receptor; endothelin A receptor antagonist in mice xenografted with the luminal A breast cancer cell line ZR75.1 induces a decrease of both bone metastases and tumor burden compared with untreated mice [190]. Interestingly, mice neonatal calvariae treated with *ET-1* expressed a lower level of Dkk-1 while they expressed a higher level of IL-6 [191]. Mixed lesions, combining both osteolytic and osteoblastic markers also exist. Like osteoblastic lesions, mixed lesions express *ET-1* but not PTHrP [187,190]. However, such lesions remain poorly understood. Furthermore, the necessity of the presence of osteoblast-like cell and microcalcifications in the primary tumor to develop such bone lesions remains unknown.

## 6. Drugs Commonly Prescribed for Osteoporosis or Rheumatoid Arthritis Treatment and Their Impact on Breast Cancer Prevalence

### 6.1. Hormone Replacement Therapies

Estrogens and progestatives were massively prescribed in the past to attenuate menopause side effects and notably the menopause-associated OP. In fact, the dramatical decrease of estrogen during menopause is responsible for a longer survival of osteoclasts. In absence of estrogens, MSCs prioritize an adipocytic differentiation rather than an osteoblastic differentiation. Estrogens are notably involved in the decrease of bone resorption-related cytokines, such as TNF-α, IL-1, and IL-6 [73]. However, as described previously, there is a dual effect of the fall of estrogen on bone tissue compared with breast tissue. If the menopause-induced increase of TNF-α, IL-1 and IL-6 results in the loss of bone mass density, hence promoting OP, it is necessary to initiate age-related lobular involution, a natural weapon against breast cancer development. In fact, as described by Hanna et al., the expression of TNF-α and IL-6 is higher in non-involuted breast compared with completely involuted breast [137]. Consequently, hormone replacement therapies (HRT) were associated with an increased risk of breast cancer development [192,193,194,195,196,197,198,199,200], and are no more prescribed in this indication nowadays.

### 6.2. Antiresorptive Drugs

#### 6.2.1. Bisphosphonates

Bisphosphonates are the first line treatment against OP in postmenopausal women. Alendronate, risedronate and zoledronate are pyrophosphate analogues. They set hydroxyapatite molecules in bone and are released because of pH changing in an area with intense osteoclasts activity. Their internalization by osteoclasts induces the activation of apoptosis, resulting in a decrease of bone resorption [201,202]. For these reasons, bisphosphonates are currently used to fight breast cancer-related hypercalcemia [203] and in metastatic breast cancer patients with bone involvement to reduce skeletal-related events and bone pain [204]. Cancer Care Ontario and the American Society of Clinical Oncology recommend the use of bisphosphonates in postmenopausal women to reduce bone metastasis recurrence and improve survival in nonmetastatic patients [205]. Many studies and reports support the use of bisphosphonates as adjuvant breast cancer therapies in postmenopausal women for their benefits on breast cancer outcomes [206,207,208,209,210,211,212]. However, some interrogations regarding the distinct effects of bisphosphonates on premenopausal and postmenopausal women for less than 5 years versus postmenopausal women for more than 5 years exist [213,214,215]. Coleman et al. observed no improvement of disease-free survival, invasive-free survival and overall survival in women older than 18 years old treated for stage II or III breast cancer with adjuvant acid zoledronic administration compared with women untreated with adjuvant zoledronic acid. However, they observed reduced bone metastases development in the adjuvant zoledronic acid treated group compared with women who did not receive adjuvant zoledronic acid. They also observed an improved invasive disease-free survival in women with established menopause treated with adjuvant zoledronic acid compared with women who did not receive adjuvant zoledronic acid [214,215]. Suarez-Almazor et al. recently reported that the administration of the recommended dose of bisphosphonates for OP in postmenopausal women older than 66 years old with breast cancer increase both overall survival and breast-cancer-specific survival after multiple adjustments [216]. The same year, van Hellemond et al. observed no effect of 3 or 6 years bisphosphonates administration before 2 to 3 years of tamoxifen treatment on postmenopausal women distant recurrence free survival, compared with women who did not receive bisphosphonates [217]. More recently, Perrone et al. observed benefits in disease-free survival in premenopausal women undergoing ovarian function suppression treated with adjuvant bisphosphonates compared with women who did not receive adjuvant bisphosphonates [218]. In vitro study demonstrated that bisphosphonate-coated bovine bone slices reduced MCF-7 luminal A and MDA-MB-231 triple-negative breast cancer cell lines abilities to adhere and proliferate compared with controls without bisphosphonate-coating [219]. Interestingly, Buranrat et al. have also demonstrated that bisphosphonates reduced migratory abilities by inducing cell cycle arrest and increasing apoptosis of the MCF-7 cell line [220]. If bisphosphonates adjuvant therapy improves breast cancer-related bone metastases outcomes, its effects on breast cancer recurrence and invasiveness remains controversial. No evidence regarding the potential role of bisphosphonates against early mammary carcinogenesis exists yet. Extensive in vitro studies should help to better decipher the direct impact of bisphosphonates on breast cancer cells.

#### 6.2.2. Selective Estrogen Receptor Modulators (SERMs)

Compared with estrogen which targets all ER-positive tissues, SERMs are capable of inducing tissue-specific ER activity. Raloxifene is a SERM used in the treatment and prevention of postmenopausal osteoporosis. Although it showed benefits relative to placebo against vertebral fractures and femoral neck bone mineral density, no benefit regarding hip fractures were statistically significant compared with placebo in randomized controlled trials in a population at risk of osteoporotic fractures [221]. More precisely, raloxifene induces a reduction of RANTES, composed notably of chemoattractant molecules, resulting in a decrease of inflammation processes in postmenopausal women [222]. In ovariectomized rats, raloxifene reversed the body weight gain, the increased leptin serum level and the decreased Ob-Rb hypothalamic expression induced by estrogen deprivation [147]. However, in women results are controversial. Some studies reported an increased leptin serum level in postmenopausal women treated with raloxifene [223,224] while Tommaselli et al. reported that raloxifene prevents postmenopausal body weight gain without modification of leptin serum level [225]. Interestingly, tamoxifen and raloxifene were reported for their association with a decrease in breast cancer risk. The Multiple Outcomes Raloxifene Evaluation (MORE) study, a multicenter, blinded, randomized placebo-controlled clinical trial reported that raloxifene reduced, by 72%, the risk of developing an invasive ER-positive breast cancer in postmenopausal women with OP during 4 years of raloxifene treatments [38]. This was then confirmed by the Continuing Outcomes Relevant to Evista (CORE) trial, which examined the effects of raloxifene versus placebo in the same cohort for 4 additional years [39]. The National Surgical Adjuvant Breast and Bowel Project (NSABP) Breast Cancer Prevention Trial (BCPT) compared both tamoxifen and raloxifene effects on invasive breast cancer risk and observed no difference between the two groups [226]. Leptin expression and Ob-Rb expression in breast cancer tissue is higher compared with non-tumoral tissue [227]. Furthermore, leptin serum level is also higher in breast cancer patients compared with healthy patients [228]. Leptin potentiates the proliferative and migratory abilities of breast cancer cells positive for ER-α [229]. Another SERM, the bazedoxifene, approved in Europe for treating OP, was described as a pure ER-α antagonist, which reduces tumor growth of both tamoxifen sensitive and resistant breast cancer cell lines xenografts. As for tamoxifen and raloxifene, it induces the proteasomal degradation of ER-α by changing its conformation in xenograft models [230]. Furthermore, Tian et al. demonstrated that bazedoxifene reduces phosphorylated-STAT3 and IL-6 mediated downstream target genes expression, but also viability, proliferation, and migration capacities of multiple triple-negative breast cancer cell lines including SUM159, MDA-MB-231, and MDA-MB-468 [231]. Interestingly, the combination of bazedoxifene and reparixin/SCH527123 targeting IL-8 had a more potent inhibition of viability, colony formation, and migration abilities on the triple-negative breast cancer cell lines SUM159 and MDA-MB-231 [232]. Bazedoxifene also induces ER-α degradation and a decrease of cell growth in wild type (WT) and D538G mutated MCF-7 luminal A breast cancer cell lines compared with untreated cell lines. However, the Y537S mutant is resistant to bazedoxifene-induced ER-α degradation but its transcription is reduced compared with untreated MCF-7 Y537S mutant [233]. In vivo experiments demonstrated that bazedoxifene inhibits the estrogens-induced ductal growth and terminal end bud formation on nude mice non-tumoral mammary tissue. Furthermore, bazedoxifene also blocked estrogens-induced tumor stimulation by increasing apoptosis and decreasing proliferation of MCF-7 cell line xenografted in nude mice [234]. In 2019, Fu et al. demonstrated in vitro that combined with the chemotherapeutic agent paclitaxel, bazedoxifene decreases cell viability, colony formation, cell migration, and potent apoptosis of ER-positive cell lines by inhibiting ER-α expression, and of triple-negative cell lines by inhibiting phosphorylated-STAT3 (Y705) and downstream targets expression [235]. In perimenopausal and postmenopausal women, combining bazedoxifene and conjugated estrogens induces favorable effects on risk biomarkers such as a decrease of mammographic fibroglandular volume and reported patients outcomes regarding menopause specific quality of life [236]. Both raloxifene and bazedoxifene represent a new hope in breast cancer prevention and personalized therapy development, including against triple negative breast cancer for which no personalized treatment exists yet.

#### 6.2.3. Denosumab

Denosumab is a human monoclonal antibody against RANKL, offered to postmenopausal OP women with a high risk of fracture, and in both women with non-metastatic breast cancer treated with aromatase inhibitor and men with non-metastatic prostate cancer treated with anti-androgenic drugs with high risk of fracture. In postmenopausal women previously treated with bisphosphonates, the risk of breast cancer development is lower in the group receiving denosumab compared with the matched group who received placebo [237]. Results of the randomized, double-blind, placebo-controlled phase 3 trial from ABCSG-18 demonstrated that adjuvant denosumab improved the disease-free survival of patients treated for early breast cancer who received adjuvant aromatase inhibitor [40]. Treatment with denosumab was also correlated with an absence of circulating tumor cells in patients with high grade invasive cancers [41]. Conversely, Coleman et al. observed no improvement in disease-related outcomes in stage II-III breast cancer patients receiving denosumab with neoadjuvant or adjuvant therapy compared with patients receiving placebo [238]. These controversies could be explained by the heterogeneity of the cohorts, especially considering the osteoimmunological disorder treated initially, but also considering the tumor phenotypes. Complementary cohort studies considering breast cancer subtypes and patients clinical characteristics should be performed before concluding. Furthermore, no in vitro study on breast cancer models were reported yet.

### 6.3. Bone Anabolic Drugs

#### 6.3.1. Parathyroid Hormone Analog

Parathyroid hormone (PTH) is produced by the parathyroid glands and regulates osteoclastic bone resorption and calcium mobilization. To do so, PTH activates TGF-ß and Wnt signaling pathways both involved in MSCs recruitment and osteoblastic differentiation [239]. Teriparatide is a recombinant form of human PTH indicated principally for severe forms of OP in postmenopausal women or in both men and women suffering from glucocorticoid-induced OP. In fact, in treated patients bone formation markers were increased by 150% and bone resorption markers by 100% in the first 3 months of treatments. However, Food and Drug Administration (FDA) stipulates that teriparatide should be used for no longer than 2 years, and it is contraindicated in patients with risk factors of osteosarcoma, Paget’s disease of bone, prior skeletal radiation, and children with open epiphyses [240]. The Forteo Patient Registry and the United States (US) postmarketing surveillance study of adult osteosarcoma and teriparatide found no incident cases of osteosarcoma after 8 and 7 years of observation in US patients receiving teriparatide treatment respectively. However, these are interim reports as the 15 years studies were not completed [241,242]. PTH is involved in bone remodeling and breast development. Intermittent PTH expression results in bone formation while continuous expression results in bone resorption. Swami et al. observed that mouse models with 4T1 murine mammary carcinoma cell line xenografted in the fat pad, pretreated then treated with intermittent PTH have the same tumor size than controls. However, the frequency of bone metastases was significantly reduced in the treated group compared with the control group. Interestingly, they also observed a decrease of 4T1 cells capacities of bone engraftment in the group pretreated with PTH compared with the control group [243]. This suggests the breast cell acquisition of PTH-related phenotypical characteristics to adhere to bone tissue. Because intermittent PTH is involved in osteoblastogenesis, it is crucial to interrogate the possibility that MSCs and breast cancer cells with mesenchymal characteristics could also develop an osteoblast-like phenotype resulting in hydroxyapatite production. Furthermore, if PTH is responsible for Wnt signaling pathway activation [239], it is crucial to examine the impact of teriparatide on involuted breast tissue that does not express SFRP1 [90], a Wnt signaling pathway antagonist. In absence of such a negative regulator, it is plausible that the overactivation of the pro-proliferative Wnt signaling pathway could result in breast hyperplasia. Even if teriparatide is only prescribed in rare severe form of OP with multiple precautions regarding patient history, it remains urgent to interrogate the impact of such treatment on both breast microcalcifications development and hyperplasia.

#### 6.3.2. Parathyroid Hormone Related Protein Analog

Parathyroid hormone related protein (PTHrP) is a paracrine regulatory hormone acting on bone forming cells by fixing PTHR1 as well as PTH. Interestingly, PTHrP is also locally produced by mammary epithelial cells to regulate cell growth and differentiation suggesting an autocrine and paracrine roles in breast microenvironment [244,245,246]. PTHrP also joins the systemic circulation to regulate maternal-to-fetal placental calcium transport [246,247]. Once more, the local effect of continuous production of PTHrP on MSCs present in the breast microenvironment has not been explored yet. In vitro, the MCF-7 luminal A breast cancer cell line overexpressing PTHrP in co-culture with murine osteoblasts and hematopoietic cells induced osteoclastogenesis [248]. Abaloparatide, a synthetic homologue of PTH (41% of homology), and PTHrP (76% of homology) is a new anabolic agent developed for OP management [249,250,251]. However, no study regarding its effect on breast tissue, breast cancer development or progression exists yet.

#### 6.3.3. Romosozumab, an Anti-Sclerostin Monoclonal Antibody

Romosozumab is an anti-sclerostin (SOST) monoclonal antibody recently proposed against severe OP. This drug has anabolic effects followed by antiresorptive effect, increasing rapidly the bone mineral density of treated patients. This treatment is administrated once a month for one year, and then stopped or changed with another antiresorptive treatment to maintain the gain in bone mass. In contrast with *SOST* mRNA, which was found in few human tissues such as heart and kidney, the sclerostin protein, a Wnt signaling antagonist was only detected in bone tissue. Sclerostin is produced by mature osteocytes to decrease osteoblastogenesis [252]. Interestingly, Hesse et al. observed that MDA-MB-231 triple-negative metastatic breast cancer cells expressed sclerostin. In addition, conditioned medium from MDA-MB-231 reduces both osteoblastic differentiation and mineralization. MDA-MB-231 mouse models treated with an anti-sclerostin antibody had less bone metastases and bone loss than the MDA-MB-231 mouse model receiving the vehicle alone [253]. However, cardiovascular serious events rate in men receiving romosozumab is higher than in men receiving the placebo [254]. This evidence was not found in postmenopausal women receiving romosozumab compared with postmenopausal women receiving the placebo [255,256]. In both men and postmenopausal women, higher levels of sclerostin were associated with aortic calcification [257,258]. However, inverse association between sclerostin serum level and aortic calcification were found in patients with chronic kidney disease after adjustment for age and cardiovascular history [259]. These controversial results demonstrate the urgent need to better understand sclerostin and romosozumab impact on soft tissue calcification, and notably on breast microcalcifications development.

### 6.4. Biological Agents Targeting Pro-Inflammatory Cytokines in Rheumatoid Arthritis

#### 6.4.1. IL-1

The transcriptomic profile of metastatic breast cancer patients overlaps systemic juvenile idiopathic arthritis, which is an IL-1 driven osteoimmunological disorder [43]. Interestingly, *IL1B* expression is increased by the addition of hydroxyapatite crystals in Hs578T triple-negative breast cancer cells culture medium [260]. Anakinra, an IL-1 receptor antagonist is a clinically approved agent used against osteoimmunological disorders, such as RA. Interestingly, Holen et al. demonstrated that the administration of anakinra in mice with subcutaneous or intravenous injection of luminal A (MCF-7) or triple negative (MDA-MB-231) breast cancer cell line reduced significantly the tumor size and the number of mice with bone metastasis compared with placebo [42]. Wu et al. also observed a decrease of tumor size after anakinra treatment administration in mice with subcutaneous injection of Hs578T [43]. More recently, Tulotta et al. confirmed the potential of IL-1B blockers in reducing breast cancer metastases by treating mice injected subcutaneously with MCF-7, MDA-MB-231 and T47D with both anakinra or canakinumab, two anti-IL1B antibodies [44]. The difficulties to test biological agents targeting cytokines ex-vivo and in vitro limit the availability of references. Progress relative to spheroids and organoids culture could help the scientific community to test the impact of such biological drugs on breast tissue.

#### 6.4.2. IL-6

Korkaya et al. demonstrated for the first time that the IL-6 receptor antibody tocilizumab, used against RA, decreases cytokines production and EMT in luminal A MCF-7 and triple-negative SUM-159 overexpressing HER2 and knocked down for PTEN (MCF-7 HER2+ PTEN- and SUM-159 HER2+ PTEN-), resulting in a decrease of tumoral aggressivity in xenograft mouse models. This is a hope for developing personalized treatment for HER2 positive tumor patients resistant to trastuzumab [45]. In 2015, Rodriguez-Barrueco et al. demonstrated that MCF10A/*Erbb2* obtained from the non-tumoral breast cell line MCF10A transformed with an oncogenic form of HER2, was sensitive to tocilizumab compared with untreated cells in both in vitro and in three-dimensional (3D) culture, and in vivo in xenograft mouse models [46]. Lin et al. demonstrated that the triple negative breast cancer cell lines MDA-MB-231 and BT-549 treated with the IL-6 receptor antibody tocilizumab dramatically lost their STAT3 activity compared with untreated cell lines [261]. The triple negative breast cancer cell line MDA-MB-231-LN co-cultured with lymphatic endothelial cells (LEC) produces IL-6, which binds the IL-6 receptor on LEC, resulting in STAT3 signaling pathway activation. In face of this evidence, Jin et al. compared MDA-MB-231-LN viability after treatment with tocilizumab and observed a decrease of cell proliferation compared with untreated cells. They also observed a decrease of tumor growth in xenograft mouse models treated with the mouse IL-6 receptor antibody [48]. More recently, Alraouji et al. demonstrated that tocilizumab inhibits IL-6 related EMT in triple negative breast cancer cell lines by inhibiting the canonical Wnt signaling pathway [47]. Another IL-6 receptor monoclonal antibody, sarilumab, improves signs and symptoms of RA comparable to tocilizumab [262,263,264]. However, no study on sarilumab in vitro effects on breast cancer cell lines have been reported yet. As mentioned previously, STAT3 is involved in the acute phase of lobular involution initiation, and the associated extracellular matrix is known to promote tumorigenesis [125,126,127,128,136]. Consequently, if tocilizumab and sarilumab seem to have potential in decreasing breast cancer cells proliferation in vitro, their impact on breast involution must be carefully studied.

#### 6.4.3. TNF-α

Two types of TNF-α targeting drugs exist; the etanercept, which targets TNF-α receptor, and infliximab, adalimumab, golimumab, and certolizumab, which directly target circulating TNF-α [265]. A phase II study of etanercept was performed in 2004 in sixteen metastatic breast cancers refractory to conventional therapy. However, no disease response was observed in the recruited cohort [266]. Hence, few studies were performed to interrogate TNF-α inhibitor therapy such as etanercept, infliximab or adalimumab safety on breast cancer patients. In women with RA treated with TNF-α inhibitor and having a history of breast cancer, no more recurrences were observed compared with other breast cancer patients who did not receive TNF-α inhibitors [267]. Mamtani et al. also observed the same results in three retrospective cohorts including women with RA and inflammatory bowel disease [268]. Chiesa Fuxench et al. explored the risk of primary breast cancer development in patients with psoriasis who received treatments including TNF-α inhibitors, without finding any changes compared with the control group of patients without psoriasis [269]. However, few meta-analyses and systematic reviews highlighted the absence of sufficient evidences to either conclude to an innocuity of TNF-α inhibitors on breast cancer development or to conclude to the existence of a relationship between the two protagonists [270,271,272]. No studies on the potential anti-tumor effect of such proteins were performed in vitro.

## 7. Conclusions

Numerous similarities between the microenvironment of bone tissue and the breast microenvironment in presence of hydroxyapatite microcalcifications have been highlighted in the present review (Table 1). Both osteoimmunological disorders and breast carcinogenesis have in common an activation of a bone resorbing microenvironment phenotype due to a dysregulation of the fragile balance between physiological and pathological inflammation. However, a bone resorbing microenvironment-like in breast tissue containing epithelial cells results in a higher aggressivity of tumoral cells, and potentially in early carcinogenesis.

Future studies should investigate both breast postlactational involution and age-related lobular involution as critical steps during which a subtle imbalance could result in both microcalcifications production and resorption, and associated chronic inflammation similar to osteoimmunological disorders. Moreover, it would be interesting to test the preventive effect of antiresorptive drugs and drugs targeting pro-inflammatory cytokines in cohorts stratified for parity history, the degree of lobular involution and the presence of microcalcifications. The safety of anabolic drugs use on women with microcalcifications should also be evaluated.

Furthermore, exploring early breast carcinogenesis as the result of a cumulative imbalance between physiological and pathological inflammation, which results in hydroxyapatite remodeling somewhat similar to bone remodeling should be further considered. A longitudinal follow-up in women after delivery and breastfeeding regarding the quality of lobular involution, the inflammatory profile and the presence of microcalcifications could help identifying patients at high risk of breast cancer development and avoid late diagnosis. Moreover, in vitro testing of both antiresorptive and drugs targeting pro-inflammatory cytokines effects on breast cell lines and their abilities to calcify could be crucial in developing microcalcifications preventive therapeutics options in the future.

## Figures and Tables

**Table 1:** Pattern of myeloid and mesenchymal lineages recruited in the osteoimmunological disorders and breast microenvironment and their cytokines production.

	PI	ARLI	Carcinogenesis	OP	RA	OA	PD
References	[72,126,129,130,131]	[90,151]	[44,151,159,160,161,162,164,165,166,167,273,274,275]	[52,53,54,55,56,57,58,70]	[30,81,82,83,84,85,86,87,88]	[56,57,58,75,76,77,276,277]	[91,92,93,94,95,96,97,99,100]
Cytokines							
IL-1	+			+	+	+	+
IL-6	+	+	+	+	+	+	+
IL-8		+					+
IL-17					+	+	
TNF-α	+	+		+	+	+	+
Leptin	+	+	+	+	+	+	+
RANKL				+	+	+	+
IL-10	-	-					
TGF-ß			-				
Others secreted protein							
SFRP1	+	+		+		+	+
Myeloid lineage							
T-cells	+	+	+	+	+	+	+
Treg					-		-
B-cells							+
Macrophages	+	+	+		+	+	+
Osteoclasts/OCL			+	+	+	+	+
Bone resorption makers							
SPP1	+		+		+		+
OPN			+		+	+	
Mesenchymal lineage							
MSCs			+		+	+	
Osteoblasts/OBL			+		+	+	+
Bone formation markers							
ALP			+				
OC			+	-	-	+	+ or =
Osteoblastogenesis markers							
RUNX2			+				

Abbreviations: PI = postlactational involution, ARLI = age-related lobular involution, OP = osteoporosis, RA = rheumatoid arthritis, OA = osteoarthritis, PD = periodontitis, IL = interleukin, TNF-α = tumor necrosis factor-alpha, RANKL = Receptor Activator of Nuclear factor Kappa-B Ligand, TGF-ß = transforming growth factor-beta, SFRP1 = secreted frizzled-related protein 1, MSCs = mesenchymal stem cells, OBL = osteoblast-like, ALP = phosphatase alkaline, OC = osteocalcin, RUNX2 = runt-related transcription factor 2.

## Abbreviations

OP	osteoporosis
IL-1	interleukin 1
TNF-α	tumor necrosis factor-alpha
RANKL	Receptor Activator of Nuclear factor Kappa-B Ligand
Ob-R	leptin receptor
MSCs	mesenchymal stem cells
SFRP1	secreted Frizzled-Related Protein 1
OA	osteoarthritis
IL-6	interleukin 6
Th17	T helper 17
IL-17	interleukin 17
ADRB2	beta-2 adrenergic receptor
RA	rheumatoid arthritis
Th1	T helper 1
Treg	T regulator
Dkk-1	dikkopf-1
TGF-ß	transforming growth factor-beta
PD	periodontitis
CD4	surface glycoprotein CD4
Th2	T helper 2
IL-8	interleukin 8
SLE	systemic lupus erythematosus
SSc	systemic sclerosis
PsA	psoriatic arthritis
BMI	body mass index
PMCA2	plasma Membrane Ca2+-ATPase
JAK	tyrosine-protein kinase JAK
STAT	signal transducer and activator of transcription
MAPK	mitogen-activated protein kinase
ERK	extracellular signal-regulated kinase
ER-α	estrogen receptor-alpha
NF-κB	Nuclear Factor-Kappa B
STAT3	Signal Transducer and Activator of Transcription 3
OPN	osteopontin
SPP1	secreted phosphoprotein 1
ER	estrogen receptor
PR	progesterone receptor
HER2	receptor tyrosine-protein kinase erbB-2
CRP	C-reactive protein
COX-2	cyclooxygenase 2
SAA1	serum amyloid 1
IL-10	interleukin 10
IDC	invasive ductal carcinoma
DCIS	ductal carcinoma in situ
RANTES	regulated upon activation, normal T-cell expressed and secreted
EMT	epithelial to mesenchymal transition
RUNX2	runt-related transcription factor 2
OBL	osteoblast-like cell
VDR	vitamin D3 receptor
BMP-2	bone morphogenetic protein 2
BMP-4	bone morphogenetic protein 4
PTX3	pentraxin-related protein PTX3
OCL	osteoclast-like giant cell
ICAM-1	intracellular adhesion molecule 1
PTHrP	parathyroid hormone-related protein
OPG	osteoprotegerin
ET-1	endothelin-1
HRT	hormone replacement therapies
SERM	selective estrogen receptor modulator
MORE	Multiple Outcomes Raloxifene Evaluation
CORE	Continuing Outcomes Relevant to Evista
NSABP	National Surgical Adjuvant Breast and Bowel Project
BCPT	Breast Cancer Prevention Trial
WT	wild type
PTHPTHrP	parathyroid hormoneparathyroid hormone related protein
FDA	Food and Drug Administration
US	United-States
SOST	sclerostin
LEC	lymphatic endothelial cells

## References

[1] Arron J.R., Choi Y. (2000). Bone versus immune system. Nature.

[2] Lorenzo J. (2020). Cytokines and Bone: Osteoimmunology. Handbook of Experimental Pharmacology.

[3] Okamoto K., Takayanagi H. (2019). Osteoimmunology. Cold Spring Harb. Perspect. Med..

[4] Ralston S.H., Schett G. (2018). Osteoimmunology. Calcif. Tissue Int..

[5] Guder C., Gravius S., Burger C., Wirtz D.C., Schildberg F.A. (2020). Osteoimmunology: A Current Update of the Interplay Between Bone and the Immune System. Front. Immunol..

[6] Tsukasaki M., Takayanagi H. (2019). Osteoimmunology: Evolving concepts in bone–immune interactions in health and disease. Nat. Rev. Immunol..

[7] Subramani R., Lakshmanaswamy R. (2017). Pregnancy and Breast Cancer. Progress in Molecular Biology and Translational Science.

[8] Bernier M.O. (2000). Breastfeeding and risk of breast cancer: A meta-analysis of published studies. Hum. Reprod. Update.

[9] Collaborative Group on Hormonal Factors in Breast Cancer (2002). Breast cancer and breastfeeding: Collaborative reanalysis of individual data from 47 epidemiological studies in 30 countries, including 50 302 women with breast cancer and 96 973 women without the disease. Lancet.

[10] Faupel-Badger J.M., Arcaro K.F., Balkam J.J., Eliassen A.H., Hassiotou F., Lebrilla C.B., Michels K.B., Palmer J.R., Schedin P., Stuebe A.M. (2013). Postpartum Remodeling, Lactation, and Breast Cancer Risk: Summary of a National Cancer Institute–Sponsored Workshop. JNCI J. Natl. Cancer Inst..

[11] Walker R., Martin C. (2007). The aged breast. J. Pathol..

[12] Gierach G.L., Patel D.A., Pfeiffer R.M., Figueroa J.D., Linville L., Papathomas D., Johnson J.M., Chicoine R.E., Herschorn S.D., Shepherd J.A. (2016). Relationship of Terminal Duct Lobular Unit Involution of the Breast with Area and Volume Mammographic Densities. Cancer Prev. Res..

[13] Ferretti G., Felici A., Cognetti F. (2007). Re: Age-Related Lobular Involution and Risk of Breast Cancer. JNCI J. Natl. Cancer Inst..

[14] Hanna M., Dumas I., Jacob S., Têtu B., Diorio C. (2015). Physical activity, mammographic density, and age-related lobular involution among premenopausal and postmenopausal women. Menopause.

[15] Henson D.E. (1993). On the possible role of involution in the natural history of breast cancer. Cancer.

[16] Radisky D.C., Visscher D.W., Frank R.D., Vierkant R.A., Winham S., Stallings-Mann M., Hoskin T.L., Nassar A., Vachon C.M., Denison L.A. (2016). Natural history of age-related lobular involution and impact on breast cancer risk. Breast Cancer Res. Treat..

[17] Ginsburg O.M., Martin L.J., Boyd N.F. (2008). Mammographic density, lobular involution, and risk of breast cancer. Br. J. Cancer.

[18] Radisky D.C., Hartmann L.C. (2009). Mammary Involution and Breast Cancer Risk: Transgenic Models and Clinical Studies. J. Mammary Gland Biol. Neoplasia.

[19] Henson D.E., Tarone R.E., Nsouli H. (2006). Lobular Involution: The Physiological Prevention of Breast Cancer. JNCI J. Natl. Cancer Inst..

[20] Henson D.E., Tarone R.E. (1994). Involution and the etiology of breast cancer. Cancer.

[21] Ghosh K., Vachon C.M., Pankratz V.S., Vierkant R.A., Anderson S.S., Brandt K.R., Visscher D.W., Reynolds C., Frost M.H., Hartmann L.C. (2010). Independent Association of Lobular Involution and Mammographic Breast Density With Breast Cancer Risk. JNCI J. Natl. Cancer Inst..

[22] Bodelon C., Oh H., Chatterjee N., Garcia-Closas M., Palakal M., Sherman M.E., Pfeiffer R.M., Geller B.M., Vacek P.M., Weaver D.L. (2017). Association between breast cancer genetic susceptibility variants and terminal duct lobular unit involution of the breast: SNPs and TDLU involution of the breast. Int. J. Cancer.

[23] Yang X.R., Figueroa J.D., Falk R.T., Zhang H., Pfeiffer R.M., Hewitt S.M., Lissowska J., Peplonska B., Brinton L., Garcia-Closas M. (2012). Analysis of terminal duct lobular unit involution in luminal A and basal breast cancers. Breast Cancer Res..

[24] Milanese T.R., Hartmann L.C., Sellers T.A., Frost M.H., Vierkant R.A., Maloney S.D., Pankratz V.S., Degnim A.C., Vachon C.M., Reynolds C.A. (2006). Age-Related Lobular Involution and Risk of Breast Cancer. JNCI J. Natl. Cancer Inst..

[25] Rominger M., Wisgickl C., Timmesfeld N. (2012). Breast Microcalcifications as Type Descriptors to Stratify risk of Malignancy: A Systematic Review and Meta-Analysis of 10665 Cases with Special Focus on Round/Punctate Microcalcifications. Fortschr Röntgenstr.

[26] Kim S.-Y., Kim H.Y., Kim E.-K., Kim M.J., Moon H.J., Yoon J.H. (2015). Evaluation of Malignancy Risk Stratification of Microcalcifications Detected on Mammography: A Study Based on the 5th Edition of BI-RADS. Ann. Surg. Oncol..

[27] Sharma T., Radosevich J.A., Pachori G., Mandal C.C. (2016). A Molecular View of Pathological Microcalcification in Breast Cancer. J. Mammary Gland Biol. Neoplasia.

[28] Bonfiglio R., Scimeca M., Toschi N., Pistolese C.A., Giannini E., Antonacci C., Ciuffa S., Tancredi V., Tarantino U., Albonici L. (2018). Radiological, Histological and Chemical Analysis of Breast Microcalcifications: Diagnostic Value and Biological Significance. J. Mammary Gland Biol. Neoplasia.

[29] Ward M.M., Alehashemi S. (2020). Risks of solid cancers in elderly persons with osteoarthritis or ankylosing spondylitis. Rheumatology.

[30] Matzelle M.M., Gallant M.A., Condon K.W., Walsh N.C., Manning C.A., Stein G.S., Lian J.B., Burr D.B., Gravallese E.M. (2012). Resolution of inflammation induces osteoblast function and regulates the Wnt signaling pathway. Arthritis Rheum..

[31] Chen H.-H., Lin C.-H., Chen D.-Y., Chao W.-C., Chen Y.-H., Hung W.-T., Chou Y.-Y., Wu Y.-D., Chen C.-C. (2019). Risk of major autoimmune diseases in female breast cancer patients: A nationwide, population-based cohort study. PLoS ONE.

[32] Bhandari B., Basyal B., Sarao M.S., Nookala V., Thein Y. (2020). Prevalence of Cancer in Rheumatoid Arthritis: Epidemiological Study Based on the National Health and Nutrition Examination Survey (NHANES). Cureus.

[33] Tian G., Liang J.-N., Wang Z.-Y., Zhou D. (2014). Breast Cancer Risk in Rheumatoid Arthritis: An Update Meta-Analysis. Biomed Res. Int..

[34] Wadström H., Pettersson A., Smedby K.E., Askling J. (2020). Risk of breast cancer before and after rheumatoid arthritis, and the impact of hormonal factors. Ann. Rheum. Dis..

[35] Shi T., Min M., Sun C., Zhang Y., Liang M., Sun Y. (2018). Periodontal disease and susceptibility to breast cancer: A meta-analysis of observational studies. J. Clin Periodontol..

[36] Sfreddo C.S., Maier J., De David S.C., Susin C., Moreira C.H.C. (2017). Periodontitis and breast cancer: A case-control study. Community Dent. Oral Epidemiol..

[37] Shao J., Wu L., Leng W.-D., Fang C., Zhu Y.-J., Jin Y.-H., Zeng X.-T. (2018). Periodontal Disease and Breast Cancer: A Meta-Analysis of 1,73,162 Participants. Front. Oncol..

[38] Cummings S.R., Eckert S., Krueger K.A., Grady D., Powles T.J., Cauley J.A., Norton L., Nickelsen T., Bjarnason N.H., Morrow M. (2000). The Effect of Raloxifene on Risk of Breast Cancer in Postmenopausal Women: Results From the MORE Randomized Trial. Obstet. Gynecol. Surv..

[39] Cauley J.A., Norton L., Lippman M.E., Eckert S., Krueger K.A., Purdie D.W., Farrerons J., Karasik A., Mellstrom D., Ng K.W. (2001). Continued Breast Cancer Risk Reduction in Postmenopausal Women Treated with Raloxifene: 4-Year Results from the MORE Trial. Breast Cancer Res. Treat..

[40] Gnant M., Pfeiler G., Steger G.G., Egle D., Greil R., Fitzal F., Wette V., Balic M., Haslbauer F., Melbinger-Zeinitzer E. (2019). Adjuvant denosumab in postmenopausal patients with hormone receptor-positive breast cancer (ABCSG-18): Disease-free survival results from a randomised, double-blind, placebo-controlled, phase 3 trial. Lancet Oncol..

[41] Vetter M., Landin J., Szczerba B.M., Castro-Giner F., Gkountela S., Donato C., Krol I., Scherrer R., Balmelli C., Malinovska A. (2018). Denosumab treatment is associated with the absence of circulating tumor cells in patients with breast cancer. Breast Cancer Res..

[42] Holen I., Lefley D.V., Francis S.E., Rennicks S., Bradbury S., Coleman R.E., Ottewell P. (2016). IL-1 drives breast cancer growth and bone metastasis *in vivo*. Oncotarget.

[43] Wu T.-C., Xu K., Martinek J., Young R.R., Banchereau R., George J., Turner J., Kim K.I., Zurawski S., Wang X. (2018). IL1 Receptor Antagonist Controls Transcriptional Signature of Inflammation in Patients with Metastatic Breast Cancer. Cancer Res..

[44] Tulotta C., Lefley D.V., Freeman K., Gregory W.M., Hanby A.M., Heath P.R., Nutter F., Wilkinson J.M., Spicer-Hadlington A.R., Liu X. (2019). Endogenous Production of IL1B by Breast Cancer Cells Drives Metastasis and Colonization of the Bone Microenvironment. Clin Cancer Res..

[45] Korkaya H., Kim G., Davis A., Malik F., Henry N.L., Ithimakin S., Quraishi A.A., Tawakkol N., D’Angelo R., Paulson A.K. (2012). Activation of an IL6 Inflammatory Loop Mediates Trastuzumab Resistance in HER2+ Breast Cancer by Expanding the Cancer Stem Cell Population. Mol. Cell.

[46] Rodriguez-Barrueco R., Yu J., Saucedo-Cuevas L.P., Olivan M., Llobet-Navas D., Putcha P., Castro V., Murga-Penas E.M., Collazo-Lorduy A., Castillo-Martin M. (2015). Inhibition of the autocrine IL-6–JAK2–STAT3–calprotectin axis as targeted therapy for HR^−^/HER2^+^ breast cancers. Genes Dev..

[47] Alraouji N.N., Al-Mohanna F.H., Ghebeh H., Arafah M., Almeer R., Al-Tweigeri T., Aboussekhra A. (2020). Tocilizumab potentiates cisplatin cytotoxicity and targets cancer stem cells in triple-negative breast cancer. Mol. Carcinog..

[48] Jin K., Pandey N.B., Popel A.S. (2018). Simultaneous blockade of IL-6 and CCL5 signaling for synergistic inhibition of triple-negative breast cancer growth and metastasis. Breast Cancer Res..

[49] Ettinger B., Pressman A., Sklarin P., Bauer D.C., Cauley J.A., Cummings S.R. (1998). Associations between Low Levels of Serum Estradiol, Bone Density, and Fractures among Elderly Women: The Study of Osteoporotic Fractures. J. Clin. Endocrinol. Metab..

[50] Cummings S.R., Ensrud K. (1998). Endogenous Hormones and the Risk of Hip and Vertebral Fractures among Older Women. N. Engl. J. Med..

[51] Garnero P., Sornay-Rendu E., Claustrat B., Delmas P.D. (2000). Biochemical Markers of Bone Turnover, Endogenous Hormones and the Risk of Fractures in Postmenopausal Women: The OFELY Study. J. Bone Miner. Res..

[52] Pacifici R., Brown C., Puscheck E., Friedrich E., Slatopolsky E., Maggio D., Mccracken R., Avioli L.V. (1991). Effect of surgical menopause and estrogen replacement on cytokine release from human blood mononuclear cells. Proc. Natl. Acad. Sci. USA.

[53] Breuil V., Ticchioni M., Testa J., Roux C.H., Ferrari P., Breittmayer J.P., Albert-Sabonnadière C., Durant J., De Perreti F., Bernard A. (2010). Immune changes in post-menopausal osteoporosis: The Immunos study. Osteoporos. Int..

[54] D’Amelio P., Grimaldi A., Di Bella S., Brianza S.Z.M., Cristofaro M.A., Tamone C., Giribaldi G., Ulliers D., Pescarmona G.P., Isaia G. (2008). Estrogen deficiency increases osteoclastogenesis up-regulating T cells activity: A key mechanism in osteoporosis. Bone.

[55] Adeel S., Singh K., Vydareny K.H., Kumari M., Shah E., Weitzmann M.N., Tangpricha V. (2013). Bone Loss in Surgically Ovariectomized Premenopausal Women Is Associated With T Lymphocyte Activation and Thymic Hypertrophy. J. Investig. Med..

[56] Dumond H., Presle N., Terlain B., Mainard D., Loeuille D., Netter P., Pottie P. (2003). Evidence for a key role of leptin in osteoarthritis. Arthritis Rheum..

[57] Wang T., He C. (2018). Pro-inflammatory cytokines: The link between obesity and osteoarthritis. Cytokine Growth Factor Rev..

[58] Astudillo P., Ríos S., Pastenes L., Pino A.M., Rodríguez J.P. (2008). Increased adipogenesis of osteoporotic human-mesenchymal stem cells (MSCs) characterizes by impaired leptin action. J. Cell. Biochem..

[59] Rehn M., Pihlajaniemi T. (1995). Identification of three N-terminal ends of type XVIII collagen chains and tissue-specific differences in the expression of the corresponding transcripts. J. Biol. Chem..

[60] Bhanot P., Brink M., Samos C.H., Hsieh J.C., Wang Y., Macke J.P., Andrew D., Nathans J., Nusse R. (1996). A new member of the frizzled family from Drosophila functions as a Wingless receptor. Nature.

[61] Finch P.W., He X., Kelley M.J., Uren A., Schaudies R.P., Popescu N.C., Rudikoff S., Aaronson S.A., Varmus H.E., Rubin J.S. (1997). Purification and molecular cloning of a secreted, Frizzled-related antagonist of Wnt action. Proc. Natl. Acad. Sci. USA.

[62] Bafico A., Gazit A., Pramila T., Finch P.W., Yaniv A., Aaronson S.A. (1999). Interaction of Frizzled Related Protein (FRP) with Wnt Ligands and the Frizzled Receptor Suggests Alternative Mechanisms for FRP Inhibition of Wnt Signaling. J. Biol. Chem..

[63] Üren A., Reichsman F., Anest V., Taylor W.G., Muraiso K., Bottaro D.P., Cumberledge S., Rubin J.S. (2000). Secreted frizzled-related protein-1 binds directly to wingless and is a biphasic modulator of wnt signaling. J. Biol. Chem..

[64] Bodine P.V.N., Zhao W., Kharode Y.P., Bex F.J., Lambert A.-J., Goad M.B., Gaur T., Stein G.S., Lian J.B., Komm B.S. (2004). The Wnt Antagonist Secreted Frizzled-Related Protein-1 Is a Negative Regulator of Trabecular Bone Formation in Adult Mice. Mol. Endocrinol..

[65] Bodine P.V.N., Billiard J., Moran R.A., Ponce-de-Leon H., McLarney S., Mangine A., Scrimo M.J., Bhat R.A., Stauffer B., Green J. (2005). The Wnt antagonist secreted frizzled-related protein-1 controls osteoblast and osteocyte apoptosis. J. Cell. Biochem..

[66] Boudin E., Fijalkowski I., Piters E., Van Hul W. (2013). The role of extracellular modulators of canonical Wnt signaling in bone metabolism and diseases. Semin. Arthritis Rheum..

[67] Monroe D.G., McGee-Lawrence M.E., Oursler M.J., Westendorf J.J. (2012). Update on Wnt signaling in bone cell biology and bone disease. Gene.

[68] Taipaleenmäki H., Abdallah B.M., AlDahmash A., Säämänen A.-M., Kassem M. (2011). Wnt signalling mediates the cross-talk between bone marrow derived pre-adipocytic and pre-osteoblastic cell populations. Exp. Cell Res..

[69] Häusler K.D., Horwood N.J., Chuman Y., Fisher J.L., Ellis J., Martin T.J., Rubin J.S., Gillespie M.T. (2004). Secreted Frizzled-Related Protein-1 Inhibits RANKL-Dependent Osteoclast Formation. J. Bone Miner. Res..

[70] Tang L., Lu W., Huang J., Tang X., Zhang H., Liu S. (2019). miR-144 promotes the proliferation and differentiation of bone mesenchymal stem cells by downregulating the expression of SFRP1. Mol. Med. Rep..

[71] Gu H., Wu L., Chen H., Huang Z., Xu J., Zhou K., Zhang Y., Chen J., Xia J., Yin X. (2019). Identification of differentially expressed microRNAs in the bone marrow of osteoporosis patients. Am. J. Transl. Res..

[72] Kothari C., Diorio C., Durocher F. (2020). The Importance of Breast Adipose Tissue in Breast Cancer. Int. J. Mol. Sci..

[73] Fontanges E., Fontana A., Delmas P. (2004). Osteoporosis and breast cancer. Jt. Bone Spine.

[74] Woodell-May J.E., Sommerfeld S.D. (2020). Role of Inflammation and the Immune System in the Progression of Osteoarthritis. J. Orthop. Res..

[75] Michou L., Numan M., Amiable N., Brown J.P. (2015). Paget’s disease of bone: An osteoimmunological disorder?. DDDT.

[76] Wojdasiewicz P., Poniatowski Ł.A., Szukiewicz D. (2014). The Role of Inflammatory and Anti-Inflammatory Cytokines in the Pathogenesis of Osteoarthritis. Mediat. Inflamm..

[77] Chow Y.Y., Chin K.-Y. (2020). The Role of Inflammation in the Pathogenesis of Osteoarthritis. Mediat. Inflamm..

[78] Elefteriou F., Ahn J.D., Takeda S., Starbuck M., Yang X., Liu X., Kondo H., Richards W.G., Bannon T.W., Noda M. (2005). Leptin regulation of bone resorption by the sympathetic nervous system and CART. Nature.

[79] Pasold J., Osterberg A., Peters K., Taipaleenmäki H., Säämänen A.-M., Vollmar B., Müller-Hilke B. (2013). Reduced expression of Sfrp1 during chondrogenesis and in articular chondrocytes correlates with osteoarthritis in STR/ort mice. Exp. Cell Res..

[80] Di Sante G., Tolusso B., Fedele A.L., Gremese E., Alivernini S., Nicolò C., Ria F., Ferraccioli G. (2015). Collagen Specific T-Cell Repertoire and HLA-DR Alleles: Biomarkers of Active Refractory Rheumatoid Arthritis. EBioMedicine.

[81] McInnes I.B., Schett G. (2011). The Pathogenesis of Rheumatoid Arthritis. N. Engl. J. Med..

[82] Pandolfi F., Franza L., Carusi V., Altamura S., Andriollo G., Nucera E. (2020). Interleukin-6 in Rheumatoid Arthritis. Int. J. Mol. Sci..

[83] McInnes I.B., Schett G. (2007). Cytokines in the pathogenesis of rheumatoid arthritis. Nat. Rev. Immunol..

[84] Taghadosi M., Samimi Z., Assar S., Salahshoor M.R., Jalili C. (2020). Plasma Leptin Does Not Reflect the Effect of High Body Mass Index on Disease Activity in Rheumatoid Arthritis. Immunol. Investig..

[85] Rodríguez J., Lafaurie G.I., Bautista-Molano W., Chila-Moreno L., Bello-Gualtero J.M., Romero-Sánchez C. (2020). Adipokines and periodontal markers as risk indicators of early rheumatoid arthritis: A cross-sectional study. Clin. Oral Investig..

[86] Jones E.A., English A., Henshaw K., Kinsey S.E., Markham A.F., Emery P., McGonagle D. (2004). Enumeration and phenotypic characterization of synovial fluid multipotential mesenchymal progenitor cells in inflammatory and degenerative arthritis. Arthritis Rheum..

[87] Djouad F., Bony C., Häupl T., Uzé G., Lahlou N., Louis-Plence P., Apparailly F., Canovas F., Rème T., Sany J. (2005). Transcriptional profiles discriminate bone marrow-derived and synovium-derived mesenchymal stem cells. Arthritis Res. Ther..

[88] Diarra D., Stolina M., Polzer K., Zwerina J., Ominsky M.S., Dwyer D., Korb A., Smolen J., Hoffmann M., Scheinecker C. (2007). Dickkopf-1 is a master regulator of joint remodeling. Nat. Med..

[89] Lee Y.-S., Lee K.-A., Yoon H.-B., Yoo S.-A., Park Y.W., Chung Y., Kim W.-U., Kang C.-Y. (2012). The Wnt inhibitor secreted Frizzled-Related Protein 1 (sFRP1) promotes human Th17 differentiation: Cellular immune response. Eur. J. Immunol..

[90] Clemenceau A., Hanna M., Ennour-Idrissi K., Burguin A., Diorio C., Durocher F. (2020). Secreted Frizzled-Related Protein 1 as a Biomarker against Incomplete Age-Related Lobular Involution and Microcalcifications’ Development. Cancers.

[91] Ponzetti M., Rucci N. (2019). Updates on Osteoimmunology: What’s New on the Cross-Talk Between Bone and Immune System. Front. Endocrinol..

[92] Brunetti G., Colucci S., Pignataro P., Coricciati M., Mori G., Cirulli N., Zallone A., Grassi F.R., Grano M. (2005). T Cells Support Osteoclastogenesis in an In Vitro Model Derived From Human Periodontitis Patients. J. Periodontol..

[93] Kawai T., Matsuyama T., Hosokawa Y., Makihira S., Seki M., Karimbux N.Y., Goncalves R.B., Valverde P., Dibart S., Li Y.-P. (2006). B and T Lymphocytes Are the Primary Sources of RANKL in the Bone Resorptive Lesion of Periodontal Disease. Am. J. Pathol..

[94] Campbell L., Millhouse E., Malcolm J., Culshaw S. (2016). T cells, teeth and tissue destruction—What do T cells do in periodontal disease?. Mol. Oral Microbiol..

[95] Cekici A., Kantarci A., Hasturk H., Van Dyke T.E. (2014). Inflammatory and immune pathways in the pathogenesis of periodontal disease: Inflammatory and immune pathways in periodontal disease. Periodontology 2000.

[96] Hoare A., Soto C., Rojas-Celis V., Bravo D. (2019). Chronic Inflammation as a Link between Periodontitis and Carcinogenesis. Mediat. Inflamm..

[97] Purwar P., Khan M.A., Mahdi A.A., Pandey S., Singh B., Dixit J., Sareen S. (2015). Salivary and Serum Leptin Concentrations in Patients With Chronic Periodontitis. J. Periodontol..

[98] Li W., Huang B., Liu K., Hou J., Meng H. (2015). Upregulated Leptin in Periodontitis Promotes Inflammatory Cytokine Expression in Periodontal Ligament Cells. J. Periodontol..

[99] Zhu J., Guo B., Gan X., Zhang L., He Y., Liu B., Chen X., Zhang S., Yu H. (2017). Association of circulating leptin and adiponectin with periodontitis: A systematic review and meta-analysis. BMC Oral Health.

[100] Li C.H., Amar S. (2007). Inhibition of SFRP1 Reduces Severity of Periodontitis. J. Dent. Res..

[101] Chan K., Clarke A.E., Ramsey-Goldman R., Foulkes W., Tessier Cloutier B., Urowitz M.B., Gladman D., Nived O., Romero-Diaz J., Petri M. (2018). Breast cancer in systemic lupus erythematosus (SLE): Receptor status and treatment. Lupus.

[102] Bernatsky S., Ramsey-Goldman R., Petri M., Urowitz M.B., Gladman D.D., Fortin P.F., Ginzler E., Romero-Diaz J., Peschken C., Jacobsen S. (2017). Breast cancer in systemic lupus. Lupus.

[103] Bernatsky S., Kale M., Ramsey-Goldman R., Gordon C., Clarke A.E. (2012). Systemic lupus and malignancies. Curr. Opin. Rheumatol..

[104] Bernatsky S., Ramsey-Goldman R., Foulkes W.D., Gordon C., Clarke A.E. (2011). Breast, ovarian, and endometrial malignancies in systemic lupus erythematosus: A meta-analysis. Br. J. Cancer.

[105] Bernatsky S., Ramsey-Goldman R., Labrecque J., Joseph L., Boivin J.-F., Petri M., Zoma A., Manzi S., Urowitz M.B., Gladman D. (2013). Cancer risk in systemic lupus: An updated international multi-centre cohort study. J. Autoimmun..

[106] Parikh-Patel A., White R.H., Allen M., Cress R. (2008). Cancer risk in a cohort of patients with systemic lupus erythematosus (SLE) in California. Cancer Causes Control.

[107] Colaci M., Giuggioli D., Vacchi C., Lumetti F., Iachetta F., Marcheselli L., Federico M., Ferri C. (2014). Breast cancer in systemic sclerosis: Results of a cross-linkage of an Italian Rheumatologic Center and a population-based Cancer Registry and review of the literature. Autoimmun. Rev..

[108] Wilton K.M., Crowson C.S., Matteson E.L. (2016). Malignancy incidence in patients with psoriatic arthritis: A comparison cohort-based incidence study. Clin. Rheumatol..

[109] Rohekar S., Tom B.D.M., Hassa A., Schentag C.T., Farewell V.T., Gladman D.D. (2008). Prevalence of malignancy in psoriatic arthritis. Arthritis Rheum..

[110] Bostoen J., Van Praet L., Brochez L., Mielants H., Lambert J. (2014). A cross-sectional study on the prevalence of metabolic syndrome in psoriasis compared to psoriatic arthritis: Metabolic syndrome in psoriatic disease. J. Eur. Acad. Derm. Venereol..

[111] Haroon M., Gallagher P., Heffernan E., FitzGerald O. (2014). High Prevalence of Metabolic Syndrome and of Insulin Resistance in Psoriatic Arthritis is Associated with the Severity of Underlying Disease. J. Rheumatol..

[112] Eder L., Harvey P., Chandran V., Rosen C.F., Dutz J., Elder J.T., Rahman P., Ritchlin C.T., Rohekar S., Hayday R. (2018). Gaps in Diagnosis and Treatment of Cardiovascular Risk Factors in Patients with Psoriatic Disease: An International Multicenter Study. J. Rheumatol..

[113] Gaudet M.M., Press M.F., Haile R.W., Lynch C.F., Glaser S.L., Schildkraut J., Gammon M.D., Douglas Thompson W., Bernstein J.L. (2011). Risk factors by molecular subtypes of breast cancer across a population-based study of women 56 years or younger. Breast Cancer Res. Treat..

[114] Turkoz F.P., Solak M., Petekkaya I., Keskin O., Kertmen N., Sarici F., Arik Z., Babacan T., Ozisik Y., Altundag K. (2013). Association between common risk factors and molecular subtypes in breast cancer patients. Breast.

[115] Protani M., Coory M., Martin J.H. (2010). Effect of obesity on survival of women with breast cancer: Systematic review and meta-analysis. Breast Cancer Res. Treat..

[116] Cross B.M., Breitwieser G.E., Reinhardt T.A., Rao R. (2014). Cellular calcium dynamics in lactation and breast cancer: From physiology to pathology. Am. J. Physiol. -Cell Physiol..

[117] VanHouten J., Sullivan C., Bazinet C., Ryoo T., Camp R., Rimm D.L., Chung G., Wysolmerski J. (2010). PMCA2 regulates apoptosis during mammary gland involution and predicts outcome in breast cancer. Proc. Natl. Acad. Sci. USA.

[118] Koktener A., Cakir B., Akin K., Kosehan D., Bayrak R., Yenidunya S. (2013). Pregnancy-like (pseudolactational) hyperplasia: Uncommon cause of microcalcifications and mass in two cases. J. Belg. Soc. Radiol..

[119] Gauger K.J., Shimono A., Crisi G.M., Schneider S. (2012). Loss of sfrp1 promotes ductal branching in the murine mammary gland. BMC Dev. Biol..

[120] Zheng X., Ning C., Dong Y., Zhao P., Li J., Fan Z., Li J., Yu Y., Mrode R., Liu J. (2017). Quantitative proteome analysis of bovine mammary gland reveals protein dynamic changes involved in peak and late lactation stages. Biochem. Biophys. Res. Commun..

[121] Aoki N. (1999). Lactation-dependent down regulation of leptin production in mouse mammary gland. Biochim. Biophys. Acta (Bba)—Gen. Subj..

[122] Basree M.M., Shinde N., Koivisto C., Cuitino M., Kladney R., Zhang J., Stephens J., Palettas M., Zhang A., Kim H.K. (2019). Abrupt involution induces inflammation, estrogenic signaling, and hyperplasia linking lack of breastfeeding with increased risk of breast cancer. Breast Cancer Res..

[123] Brann W.D., Wade M.F., Dhandapani K.M., Mahesh V.B., Buchanan C.D. (2001). Leptin and reproduction. Steroids.

[124] Diano S., Kalra S.P., Sakamoto H., Horvath T.L. (1998). Leptin receptors in estrogen receptor-containing neurons of the female rat hypothalamus. Brain Res..

[125] Pensa S., Watson C.J., Poli V. (2009). Stat3 and the Inflammation/Acute Phase Response in Involution and Breast Cancer. J. Mammary Gland Biol. Neoplasia.

[126] Poli V. (1998). The Role of C/EBP Isoforms in the Control of Inflammatory and Native Immunity Functions. J. Biol. Chem..

[127] Chapman R.S., Lourenco P.C., Tonner E., Flint D.J., Selbert S., Takeda K., Akira S., Clarke A.R., Watson C.J. (1999). Suppression of epithelial apoptosis and delayed mammary gland involution in mice with a conditional knockout of Stat3. Genes Dev..

[128] Humphreys R.C., Bierie B., Zhao L., Raz R., Levy D., Hennighausen L. (2002). Deletion of Stat3 Blocks Mammary Gland Involution and Extends Functional Competence of the Secretory Epithelium in the Absence of Lactogenic Stimuli. Endocrinology.

[129] Martinson H.A., Jindal S., Durand-Rougely C., Borges V.F., Schedin P. (2015). Wound healing-like immune program facilitates postpartum mammary gland involution and tumor progression: Immune cells in postpartum involution and breast cancer. Int. J. Cancer.

[130] Prokesch A., Smorlesi A., Perugini J., Manieri M., Ciarmela P., Mondini E., Trajanoski Z., Kristiansen K., Giordano A., Bogner-Strauss J.G. (2014). Molecular Aspects of Adipoepithelial Transdifferentiation in Mouse Mammary Gland: Adipoepithelial Transdifferentiation. Stem Cells.

[131] Rittling R., Novick E. (1997). Osteopontin Expression in Mammary Gland Development and Tumorigenesis. Cell Growth Differ..

[132] Huan J.-L., Xing L., Qin X.-J., Gao Z.-G., Pan X.-F., Zhao Z.-D. (2012). Expression and Clinical Significance of Osteopontin in Calcified Breast Tissue. Asian Pac. J. Cancer Prev..

[133] Bellahc A. (1995). Increased Expression of Osteonectin and Osteopontin, Two Bone Matrix Proteins, in Human Breast Cancer. Am. J. Pathol..

[134] Kothari C., Ouellette G., Labrie Y., Jacob S., Diorio C., Durocher F. (2018). Identification of a gene signature for different stages of breast cancer development that could be used for early diagnosis and specific therapy. Oncotarget.

[135] Rizwan A., Paidi S.K., Zheng C., Cheng M., Barman I., Glunde K. (2018). Mapping the genetic basis of breast microcalcifications and their role in metastasis. Sci. Rep..

[136] McDaniel S.M., Rumer K.K., Biroc S.L., Metz R.P., Singh M., Porter W., Schedin P. (2006). Remodeling of the Mammary Microenvironment after Lactation Promotes Breast Tumor Cell Metastasis. Am. J. Pathol..

[137] Hanna M., Dumas I., Orain M., Jacob S., Têtu B., Sanschagrin F., Bureau A., Poirier B., Diorio C. (2017). Association between local inflammation and breast tissue age-related lobular involution among premenopausal and postmenopausal breast cancer patients. PLoS ONE.

[138] Veeck J., Niederacher D., An H., Klopocki E., Wiesmann F., Betz B., Galm O., Camara O., Dürst M., Kristiansen G. (2006). Aberrant methylation of the Wnt antagonist SFRP1 in breast cancer is associated with unfavourable prognosis. Oncogene.

[139] Huth L., Rose M., Kloubert V., Winkens W., Schlensog M., Hartmann A., Knüchel R., Dahl E. (2014). BDNF Is Associated with SFRP1 Expression in Luminal and Basal-Like Breast Cancer Cell Lines and Primary Breast Cancer Tissues: A Novel Role in Tumor Suppression?. PLoS ONE.

[140] Yang Y., Xing Y., Liang C., Hu L., Xu F., Chen Y. (2015). Crucial microRNAs and genes of human primary breast cancer explored by microRNA-mRNA integrated analysis. Tumor Biol..

[141] Wang Z., Li R., He Y., Huang S. (2018). Effects of secreted frizzled-related protein 1 on proliferation, migration, invasion, and apoptosis of colorectal cancer cells. Cancer Cell Int..

[142] Gregory K.J., Schneider S.S. (2015). Estrogen-mediated signaling is differentially affected by the expression levels of Sfrp1 in mammary epithelial cells: Estrogen signaling and *Sfrp1* expression. Cell Biol. Int..

[143] Dahl E., Wiesmann F., Woenckhaus M., Stoehr R., Wild P.J., Veeck J., Knüchel R., Klopocki E., Sauter G., Simon R. (2007). Frequent loss of SFRP1 expression in multiple human solid tumours: Association with aberrant promoter methylation in renal cell carcinoma. Oncogene.

[144] Gregory K.J., Roberts A.L., Conlon E.M., Mayfield J.A., Hagen M.J., Crisi G.M., Bentley B.A., Kane J.J., Makari-Judson G., Mason H.S. (2019). Gene expression signature of atypical breast hyperplasia and regulation by SFRP1. Breast Cancer Res..

[145] Chiu Y.-C., Wang L.-J., Hsiao T.-H., Chuang E.Y., Chen Y. (2017). Genome-wide identification of key modulators of gene-gene interaction networks in breast cancer. BMC Genom..

[146] Bernemann C., Hülsewig C., Ruckert C., Schäfer S., Blümel L., Hempel G., Götte M., Greve B., Barth P.J., Kiesel L. (2014). Influence of secreted frizzled receptor protein 1 (SFRP1) on neoadjuvant chemotherapy in triple negative breast cancer does not rely on WNT signaling. Mol. Cancer.

[147] Meli R., Pacilio M., Raso G.M., Esposito E., Coppola A., Nasti A., Di Carlo C., Nappi C., Di Carlo R. (2004). Estrogen and Raloxifene Modulate Leptin and Its Receptor in Hypothalamus and Adipose Tissue from Ovariectomized Rats. Endocrinology.

[148] Naseem M., Murray J., Hilton J.F., Karamchandani J., Muradali D., Faragalla H., Polenz C., Han D., Bell D.C., Brezden-Masley C. (2015). Mammographic microcalcifications and breast cancer tumorigenesis: A radiologic-pathologic analysis. BMC Cancer.

[149] Siegel R.L., Miller K.D., Jemal A. (2020). Cancer statistics, 2020. CA Cancer J. Clin..

[150] Quigley D.A., Tahiri A., Lüders T., Riis M.H., Balmain A., Børresen-Dale A.-L., Bukholm I., Kristensen V. (2017). Age, estrogen, and immune response in breast adenocarcinoma and adjacent normal tissue. OncoImmunology.

[151] Hanna M., Dumas I., Orain M., Jacob S., Têtu B., Sanschagrin F., Bureau A., Poirier B., Diorio C. (2017). Association between expression of inflammatory markers in normal breast tissue and mammographic density among premenopausal and postmenopausal women. Menopause.

[152] Dunn G.P., Bruce A.T., Ikeda H., Old L.J., Schreiber R.D. (2002). Cancer immunoediting: From immunosurveillance to tumor escape. Nat. Immunol..

[153] Desmedt C., Salgado R., Fornili M., Pruneri G., Van den Eynden G., Zoppoli G., Rothé F., Buisseret L., Garaud S., Willard-Gallo K. (2018). Immune Infiltration in Invasive Lobular Breast Cancer. JNCI J. Natl. Cancer Inst..

[154] Autenshlyus A.I., Kunts T.A., Karpukhina K.V., Mikhaylova E.S., Varaksin N.A., Marinkin I.O., Lyakhovich V.V. (2016). Cytokine pattern of the breast tumor supernatant. Dokl. Biol. Sci.

[155] Clemenceau A., Diorio C., Durocher F. (2020). Role of Secreted Frizzled-Related Protein 1 in Early Mammary Gland Tumorigenesis and Its Regulation in Breast Microenvironment. Cells.

[156] Ercan C., van Diest P.J., Vooijs M. (2011). Mammary Development and Breast Cancer: The Role of Stem Cells. CMM.

[157] Macias H., Hinck L. (2012). Mammary gland development: Mammary gland development. Wires Dev. Biol..

[158] Reya T., Clevers H. (2005). Wnt signalling in stem cells and cancer. Nature.

[159] Tan C.-C., Li G.-X., Tan L.-D., Du X., Li X.-Q., He R., Wang Q.-S., Feng Y.-M. (2016). Breast cancer cells obtain an osteomimetic feature via epithelial-mesenchymal transition that have undergone BMP2/RUNX2 signaling pathway induction. Oncotarget.

[160] Scimeca M., Urbano N., Bonfiglio R., Schillaci O., Bonanno E. (2018). Breast osteoblast-like cells: A new biomarker for the management of breast cancer. Br. J. Cancer.

[161] Scimeca M., Bonfiglio R., Menichini E., Albonici L., Urbano N., De Caro M.T., Mauriello A., Schillaci O., Gambacurta A., Bonanno E. (2019). Microcalcifications Drive Breast Cancer Occurrence and Development by Macrophage-Mediated Epithelial to Mesenchymal Transition. Int. J. Mol. Sci..

[162] Valentin-Opran A., Eilon G., Saez S., Mundy G.R. (1985). Estrogens and antiestrogens stimulate release of bone resorbing activity by cultured human breast cancer cells. J. Clin. Investig..

[163] Vicard E., Valentin-Opran A., Chenu C., Delmas P.D., Meunier P.J., Saez S. (1986). Androgens increase osteoblast-stimulating activity of human breast cancer cells in vitro. J. Steroid Biochem..

[164] Cox R.F., Hernandez-Santana A., Ramdass S., McMahon G., Harmey J.H., Morgan M.P. (2012). Microcalcifications in breast cancer: Novel insights into the molecular mechanism and functional consequence of mammary mineralisation. Br. J. Cancer.

[165] Cox R.F., Morgan M.P. (2013). Microcalcifications in breast cancer: Lessons from physiological mineralization. Bone.

[166] Agnantis N.T., Rosen P.P. (1979). Mammary Carcinoma with Osteoclast-like Giant Cells: A Study of Eight Cases with Follow-up Data. Am. J. Clin. Pathol..

[167] Athanasou N., Wells C., Quinn J., Ferguson D., Heryet A., McGee J. (1989). The origin and nature of stromal osteoclast-like multinucleated giant cells in breast carcinoma: Implications for tumour osteolysis and macrophage biology. Br. J. Cancer.

[168] Zagelbaum N.K., Ward M.F., Okby N., Karpoff H. (2016). Invasive ductal carcinoma of the breast with osteoclast-like giant cells and clear cell features: A case report of a novel finding and review of the literature. World J. Surg. Oncol..

[169] Xu Z., Gu J., Zhang S., Zhang Z., Fang W. (2019). Leiomyosarcoma with osteoclast-like (LMS-OGC) giant cells the breast: A report of a rare case. Thorac. Cancer.

[170] Cai N., Koizumi J., Vazquez M. (2005). Mammary carcinoma with osteoclast-like giant cells: A study of four cases and a review of literature. Diagn. Cytopathol..

[171] Richter G., Uleer C., Noesselt T. (2011). Multifocal invasive ductal breast cancer with osteoclast-like giant cells: A case report. J. Med. Case Rep..

[172] Cozzolino I., Ciancia G., Limite G., Di Micco R., Varone V., Cortese A., Vatrella A., Di Crescenzo V., Zeppa P. (2014). Neuroendocrine differentiation in breast carcinoma with osteoclast-like giant cells. Report of a case and review of the literature. Int. J. Surg..

[173] Peña-Jaimes L., González-García I., Reguero-Callejas M.E., Pinilla-Pagnon I., Pérez-Mies B., Albarrán-Artahona V., Martínez-Jañez N., Rosa-Rosa J.M., Palacios J. (2018). Pleomorphic lobular carcinoma of the breast with osteoclast-like giant cells: A case report and review of the literature. Diagn. Pathol..

[174] Krishnan C., Longacre T.A. (2006). Ductal carcinoma in situ of the breast with osteoclast-like giant cells. Hum. Pathol..

[175] Romics L., Mallon E.A., Reid R., Cordiner C.M., Doughty J.C. (2009). Osteoclast-like giant cell tumor arising in the soft tissue of the breast: Report of a case. Surg. Today.

[176] (1984). Mammary carcinoma with osteoclast-like giant cells: Additional observations on six cases. Cancer.

[177] Ohashi R., Hayama A., Matsubara M., Watarai Y., Sakatani T., Naito Z., Shimizu A. (2018). Breast carcinoma with osteoclast-like giant cells: A cytological-pathological correlation with a literature review. Ann. Diagn. Pathol..

[178] Ohashi R., Yanagihara K., Namimatsu S., Sakatani T., Takei H., Naito Z., Shimizu A. (2018). Osteoclast-like giant cells in invasive breast cancer predominantly possess M2-macrophage phenotype. Pathol. Res. Pract..

[179] Tsai C.-F., Chen J.-H., Wu C.-T., Chang P.-C., Wang S.-L., Yeh W.-L. (2019). Induction of osteoclast-like cell formation by leptin-induced soluble intercellular adhesion molecule secreted from cancer cells. Adv. Med. Oncol..

[180] Guise T.A., Yin J.J., Taylor S.D., Kumagai Y., Dallas M., Boyce B.F., Yoneda T., Mundy G.R. (1996). Evidence for a causal role of parathyroid hormone-related protein in the pathogenesis of human breast cancer-mediated osteolysis. J. Clin. Investig..

[181] Powell G.J., Southby J., Danks J.A., Stillwell R.G., Hayman J.A., Henderson M.A., Bennett R.C., Martin T.J. (1991). Localization of Parathyroid Hormone-related Protein in Breast Cancer Metastases: Increased Incidence in Bone Compared with Other Sites. Cancer Res..

[182] Taipaleenmäki H., Farina N.H., van Wijnen A.J., Stein J.L., Hesse E., Stein G.S., Lian J.B. (2016). Antagonizing miR-218-5p attenuates Wnt signaling and reduces metastatic bone disease of triple negative breast cancer cells. Oncotarget.

[183] Bendre M.S., Montague D.C., Peery T., Akel N.S., Gaddy D., Suva L.J. (2003). Interleukin-8 stimulation of osteoclastogenesis and bone resorption is a mechanism for the increased osteolysis of metastatic bone disease. Bone.

[184] Kovacheva M., Zepp M., Schraad M., Berger S., Berger M.R. (2019). Conditional Knockdown of Osteopontin Inhibits Breast Cancer Skeletal Metastasis. Int. J. Mol. Sci..

[185] Mariz K., Ingolf J.-B., Daniel H., Teresa N.J., Erich-Franz S. (2015). The Wnt inhibitor dickkopf-1: A link between breast cancer and bone metastases. Clin. Exp. Metastasis.

[186] Voorzanger-Rousselot N., Goehrig D., Journe F., Doriath V., Body J.J., Clézardin P., Garnero P. (2007). Increased Dickkopf-1 expression in breast cancer bone metastases. Br. J. Cancer.

[187] Guise T.A., Kozlow W.M., Heras-Herzig A., Padalecki S.S., Yin J.J., Chirgwin J.M. (2005). Molecular Mechanisms of Breast Cancer Metastases to Bone. Clin. Breast Cancer.

[188] Fang J., Xu Q. (2015). Differences of osteoblastic bone metastases and osteolytic bone metastases in clinical features and molecular characteristics. Clin. Transl. Oncol..

[189] Nelson J.B., Hedican S.P., George D.J., Reddi A.H., Piantadosi S., Eisenberger M.A., Simons J.W. (1995). Identification of endothelin–1 in the pathophysiology of metastatic adenocarcinoma of the prostate. Nat. Med..

[190] Yin J.J., Mohammad K.S., Kakonen S.M., Harris S., Wu-Wong J.R., Wessale J.L., Padley R.J., Garrett I.R., Chirgwin J.M., Guise T.A. (2003). A causal role for endothelin-1 in the pathogenesis of osteoblastic bone metastases. Proc. Natl. Acad. Sci. USA.

[191] Clines G.A., Mohammad K.S., Bao Y., Stephens O.W., Suva L.J., Shaughnessy J.D., Fox J.W., Chirgwin J.M., Guise T.A. (2007). Dickkopf Homolog 1 Mediates Endothelin-1-Stimulated New Bone Formation. Mol. Endocrinol..

[192] Beral V. (2003). Million Women Study Collaborators. Breast cancer and hormone-replacement therapy in the Million Women Study. Lancet.

[193] Newcomb P.A., Titus-Ernstoff L., Egan K.M., Trentham-Dietz A., Baron J.A., Storer B.E., Willett W.C., Stampfer M.J. (2002). Postmenopausal Estrogen and Progestin Use in Relation to Breast Cancer Risk. Cancer Epidemiol. Biomark. Prev..

[194] Stahlberg C., Pedersen A.T., Lynge E., Andersen Z.J., Keiding N., Hundrup Y.A., Obel E.B., Ottesen B. (2004). Increased risk of breast cancer following different regimens of hormone replacement therapy frequently used in Europe. Int. J. Cancer.

[195] Fournier A., Berrino F., Clavel-Chapelon F. (2007). Unequal risks for breast cancer associated with different hormone replacement therapies: Results from the E3N cohort study. Breast Cancer Res. Treat..

[196] Ewertz M., Mellemkjaer L., Poulsen A.H., Friis S., Sørensen H.T., Pedersen L., McLaughlin J.K., Olsen J.H. (2005). Hormone use for menopausal symptoms and risk of breast cancer. A Danish cohort study. Br. J. Cancer.

[197] Lee S., Kolonel L., Wilkens L., Wan P., Henderson B., Pike M. (2006). Postmenopausal hormone therapy and breast cancer risk: The multiethnic cohort. Int. J. Cancer.

[198] Magnusson C., Baron J.A., Correia N., Bergström R., Adami H.O., Persson I. (1999). Breast-cancer risk following long-term oestrogen- and oestrogen-progestin-replacement therapy. Int. J. Cancer.

[199] Chen W.Y., Manson J.E., Hankinson S.E., Rosner B., Holmes M.D., Willett W.C., Colditz G.A. (2006). Unopposed Estrogen Therapy and the Risk of Invasive Breast Cancer. Arch. Intern. Med..

[200] Collaborative Group on Hormonal Factors in Breast Cancer (1997). Breast cancer and hormone replacement therapy: Collaborative reanalysis of data from 51 epidemiological studies of 52 705 women with breast cancer and 108 411 women without breast cancer. Lancet.

[201] Ishtiaq S., Fogelman I., Hampson G. (2015). Treatment of post-menopausal osteoporosis: Beyond bisphosphonates. J. Endocrinol. Investig..

[202] Ewelina B., Fengfeng C., Marcus V. (2017). Bone targeted therapies in advanced breast cancer. Swiss Med. Wkly..

[203] Elomaa I., Blomqvist C., Gröhn P., Porkaa L., Kairento A.L., Selander K., Lamberg-Allardt C., Holmström T. (1983). Long-term controlled trial with diphosphonate in patients with osteolytic bone metastases. Lancet.

[204] Coleman R. (2020). Bisphosphonates and breast cancer—From cautious palliation to saving lives. Bone.

[205] Dhesy-Thind S., Fletcher G.G., Blanchette P.S., Clemons M.J., Dillmon M.S., Frank E.S., Gandhi S., Gupta R., Mates M., Moy B. (2017). Use of Adjuvant Bisphosphonates and Other Bone-Modifying Agents in Breast Cancer: A Cancer Care Ontario and American Society of Clinical Oncology Clinical Practice Guideline. JCO.

[206] Gnant M. (2009). The evolving role of zoledronic acid in early breast cancer. OTT.

[207] Gnant M. (2014). Role of bisphosphonates in postmenopausal women with breast cancer. Cancer Treat. Rev..

[208] Gnant M. (2012). Zoledronic Acid in the Treatment of Early-Stage Breast Cancer: Is There a Final Verdict?. Curr. Oncol. Rep..

[209] Gnant M. (2011). Zoledronic acid in breast cancer: Latest findings and interpretations. Ther. Adv. Med. Oncol..

[210] Gnant M., Mlineritsch B., Stoeger H., Luschin-Ebengreuth G., Heck D., Menzel C., Jakesz R., Seifert M., Hubalek M., Pristauz G. (2011). Adjuvant endocrine therapy plus zoledronic acid in premenopausal women with early-stage breast cancer: 62-month follow-up from the ABCSG-12 randomised trial. Lancet Oncol..

[211] Gnant M., Mlineritsch B., Stoeger H., Luschin-Ebengreuth G., Knauer M., Moik M., Jakesz R., Seifert M., Taucher S., Bjelic-Radisic V. (2015). Zoledronic acid combined with adjuvant endocrine therapy of tamoxifen versus anastrozol plus ovarian function suppression in premenopausal early breast cancer: Final analysis of the Austrian Breast and Colorectal Cancer Study Group Trial 12. Ann. Oncol..

[212] Gnant M., Mlineritsch B., Luschin-Ebengreuth G., Kainberger F., Kässmann H., Piswanger-Sölkner J.C., Seifert M., Ploner F., Menzel C., Dubsky P. (2008). Adjuvant endocrine therapy plus zoledronic acid in premenopausal women with early-stage breast cancer: 5-year follow-up of the ABCSG-12 bone-mineral density substudy. Lancet Oncol..

[213] Coleman R.E., Marshall H., Cameron D., Dodwell D., Burkinshaw R., Keane M., Gil M., Houston S.J., Grieve R.J., Barrett-Lee P.J. (2011). Breast-Cancer Adjuvant Therapy with Zoledronic Acid. N. Engl. J. Med..

[214] Coleman R., Cameron D., Dodwell D., Bell R., Wilson C., Rathbone E., Keane M., Gil M., Burkinshaw R., Grieve R. (2014). Adjuvant zoledronic acid in patients with early breast cancer: Final efficacy analysis of the AZURE (BIG 01/04) randomised open-label phase 3 trial. Lancet Oncol..

[215] Coleman R.E., Collinson M., Gregory W., Marshall H., Bell R., Dodwell D., Keane M., Gil M., Barrett-Lee P., Ritchie D. (2018). Benefits and risks of adjuvant treatment with zoledronic acid in stage II/III breast cancer. 10 years follow-up of the AZURE randomized clinical trial (BIG 01/04). J. Bone Oncol..

[216] Suarez-Almazor M.E., Herrera R., Lei X., Chavez-MacGregor M., Zhao H., Giordano S.H. (2020). Survival in older women with early stage breast cancer receiving low-dose bisphosphonates or denosumab. Cancer.

[217] van Hellemond I.E.G., Smorenburg C.H., Peer P.G.M., Swinkels A.C.P., Seynaeve C.M., van der Sangen M.J.C., Kroep J.R., de Graaf H., Honkoop A.H., Erdkamp F.L. (2020). Breast cancer outcome in relation to bone mineral density and bisphosphonate use: A sub-study of the DATA trial. Breast Cancer Res. Treat..

[218] Perrone F., De Laurentiis M., De Placido S., Orditura M., Cinieri S., Riccardi F., Ribecco A.S., Putzu C., Del Mastro L., Rossi E. (2019). Adjuvant zoledronic acid and letrozole plus ovarian function suppression in premenopausal breast cancer: HOBOE phase 3 randomised trial. Eur. J. Cancer.

[219] Park Y.-E., Bava U., Lin J., Cornish J., Naot D., Reid I.R. (2019). Bone-Bound Bisphosphonates Inhibit Proliferation of Breast Cancer Cells. Calcif. Tissue Int..

[220] Buranrat B., Bootha S. (2019). Antiproliferative and antimigratory activities of bisphosphonates in human breast cancer cell line MCF-7. Oncol. Lett..

[221] Simpson E.L., Martyn-St James M., Hamilton J., Wong R., Gittoes N., Selby P., Davis S. (2020). Clinical effectiveness of denosumab, raloxifene, romosozumab, and teriparatide for the prevention of osteoporotic fragility fractures: A systematic review and network meta-analysis. Bone.

[222] Christodoulakos G.E., Lambrinoudaki I.V., Economou E.V., Papadias C., Vitoratos N., Panoulis C.P., Kouskouni E.E., Vlachou S.A., Creatsas G.C. (2007). Circulating chemoattractants RANTES, negatively related to endogenous androgens, and MCP-1 are differentially suppressed by hormone therapy and raloxifene. Atherosclerosis.

[223] Sebastián-Ochoa A., Fernández-García D., Reyes-García R., Mezquita-Raya P., Rozas-Moreno P., Alonso-Garcia G., Muñoz-Torres M. (2012). Adiponectin and leptin serum levels in osteoporotic postmenopausal women treated with raloxifene or alendronate. Menopause J. N. Am. Menopause Soc..

[224] Lambrinoudaki I.V., Christodoulakos G.E., Economou E.V., Vlachou S.A., Panoulis C.P., Alexandrou A.P., Kouskouni E.E., Creatsas G.C. (2008). Circulating leptin and ghrelin are differentially influenced by estrogen/progestin therapy and raloxifene. Maturitas.

[225] Tommaselli G.A., Di Carlo C., Di Spiezio Sardo A., Bifulco G., Cirillo D., Guida M., Capasso R., Nappi C. (2006). Serum leptin levels and body composition in postmenopausal women treated with tibolone and raloxifene. Menopause.

[226] Corson S.L. (2006). Effects of tamoxifen vs raloxifene on the risk of developing invasive breast cancer and other disease outcomes. J. Minim. Invasive Gynecol..

[227] Garofalo C. (2006). Increased Expression of Leptin and the Leptin Receptor as a Marker of Breast Cancer Progression: Possible Role of Obesity-Related Stimuli. Clin. Cancer Res..

[228] Niu J., Jiang L., Guo W., Shao L., Liu Y., Wang L. (2013). The Association between Leptin Level and Breast Cancer: A Meta-Analysis. PLoS ONE.

[229] Haque I., Ghosh A., Acup S., Banerjee S., Dhar K., Ray A., Sarkar S., Kambhampati S., Banerjee S.K. (2018). Leptin-induced ER-α-positive breast cancer cell viability and migration is mediated by suppressing CCN5-signaling via activating JAK/AKT/STAT-pathway. BMC Cancer.

[230] Wardell S.E., Nelson E.R., Chao C.A., McDonnell D.P. (2013). Bazedoxifene Exhibits Antiestrogenic Activity in Animal Models of Tamoxifen-Resistant Breast Cancer: Implications for Treatment of Advanced Disease. Clin. Cancer Res..

[231] Tian J., Chen X., Fu S., Zhang R., Pan L., Cao Y., Wu X., Xiao H., Lin H.-J., Lo H.-W. (2019). Bazedoxifene is a novel IL-6/GP130 inhibitor for treating triple-negative breast cancer. Breast Cancer Res. Treat..

[232] Fu S., Lin J. (2018). Blocking Interleukin-6 and Interleukin-8 Signaling Inhibits Cell Viability, Colony-forming Activity, and Cell Migration in Human Triple-negative Breast Cancer and Pancreatic Cancer Cells. Anticancer Res..

[233] Fanning S.W., Jeselsohn R., Dharmarajan V., Mayne C.G., Karimi M., Buchwalter G., Houtman R., Toy W., Fowler C.E., Han R. (2018). The SERM/SERD bazedoxifene disrupts ESR1 helix 12 to overcome acquired hormone resistance in breast cancer cells. eLife.

[234] Santen R.J., Song Y., Wang J., Yue W. (2017). Preclinical breast effects of a tissue selective estrogen complex (TSEC) including conjugated estrogen with bazedoxifene. J. Steroid Biochem. Mol. Biol..

[235] Fu S., Chen X., Lo H.-W., Lin J. (2019). Combined bazedoxifene and paclitaxel treatments inhibit cell viability, cell migration, colony formation, and tumor growth and induce apoptosis in breast cancer. Cancer Lett..

[236] Fabian C.J., Nye L., Powers K.R., Nydegger J.L., Kreutzjans A.L., Phillips T.A., Metheny T., Winblad O., Zalles C.M., Hagan C.R. (2019). Effect of Bazedoxifene and Conjugated Estrogen (Duavee) on Breast Cancer Risk Biomarkers in High-Risk Women: A Pilot Study. Cancer Prev. Res..

[237] Giannakeas V., Cadarette S.M., Ban J.K., Lipscombe L., Narod S.A., Kotsopoulos J. (2018). Denosumab and breast cancer risk in postmenopausal women: A population-based cohort study. Br. J. Cancer.

[238] Coleman R., Finkelstein D.M., Barrios C., Martin M., Iwata H., Hegg R., Glaspy J., Periañez A.M., Tonkin K., Deleu I. (2020). Adjuvant denosumab in early breast cancer (D-CARE): An international, multicentre, randomised, controlled, phase 3 trial. Lancet Oncol..

[239] Crane J.L., Cao X. (2014). Bone marrow mesenchymal stem cells and TGF-β signaling in bone remodeling. J. Clin. Investig..

[240] Capriani C., Irani D., Bilezikian J.P. (2012). Safety of osteoanabolic therapy: A decade of experience. J. Bone Miner. Res..

[241] Gilsenan A., Harding A., Kellier-Steele N., Harris D., Midkiff K., Andrews E. (2018). The Forteo Patient Registry linkage to multiple state cancer registries: Study design and results from the first 8 years. Osteoporos. Int..

[242] Andrews E.B., Gilsenan A.W., Midkiff K., Sherrill B., Wu Y., Mann B.H., Masica D. (2012). The US postmarketing surveillance study of adult osteosarcoma and teriparatide: Study design and findings from the first 7 years. J. Bone Miner. Res..

[243] Swami S., Johnson J., Bettinson L.A., Kimura T., Zhu H., Albertelli M.A., Johnson R.W., Wu J.Y. (2017). Prevention of breast cancer skeletal metastases with parathyroid hormone. JCI Insight.

[244] Grill V., Hillary J., Ho P.M.W., Law F.M.K., Macisaac R.J., Maclsaac I.A., Moseley J.M., Martin T.J. (1992). Parathyroid hormone-related protein: A possible endocrine function in lactation. Clin. Endocrinol..

[245] Rakopoulos M., Vargas S.J., Gillespie M.T., Ho P.W., Diefenbach-Jagger H., Leaver D.D., Grill V., Moseley J.M., Danks J.A., Martin T.J. (1993). Production of parathyroid hormone-related protein by the rat mammary gland in pregnancy and lactation. Am. J. Physiol..

[246] Martin T.J., Johnson R.W. (2019). Multiple actions of parathyroid hormone-related protein in breast cancer bone metastasis. Br. J. Pharm..

[247] Kovacs C.S., Lanske B., Hunzelman J.L., Guo J., Karaplis A.C., Kronenberg H.M. (1996). Parathyroid hormone-related peptide (PTHrP) regulates fetal-placental calcium transport through a receptor distinct from the PTH/PTHrP receptor. Proc. Natl. Acad. Sci. USA.

[248] Thomas R.J., Guise T.A., Yin J.J., Elliott J., Horwood N.J., Martin T.J., Gillespie M.T. (1999). Breast Cancer Cells Interact with Osteoblasts to Support Osteoclast Formation. Endocrinology.

[249] Sleeman A., Clements J.N. (2019). Abaloparatide: A new pharmacological option for osteoporosis. Am. J. Health Syst. Pharm..

[250] Boyce E.G., Mai Y., Pham C. (2018). Abaloparatide: Review of a next-generation parathyroid hormone agonist. Ann. Pharm..

[251] Tella S.H., Kommalapati A., Correa R. (2017). Profile of abaloparatide and its potential in the treatment of postmenopausal osteoporosis. Cureus.

[252] Gkotzamanidou M., Dimopoulos M.A., Kastritis E., Christoulas D., Moulopoulos L.A., Terpos E. (2012). Sclerostin: A possible target for the management of cancer-induced bone disease. Expert Opin. Ther. Targets.

[253] Hesse E., Schröder S., Brandt D., Pamperin J., Saito H., Taipaleenmäki H. (2019). Sclerostin inhibition alleviates breast cancer–induced bone metastases and muscle weakness. JCI Insight.

[254] Lewiecki E.M., Blicharski T., Goemaere S., Lippuner K., Meisner P.D., Miller P.D., Miyauchi A., Maddox J., Chen L., Horlait S. (2018). A Phase III Randomized Placebo-Controlled Trial to Evaluate Efficacy and Safety of Romosozumab in Men With Osteoporosis. J. Clin. Endocrinol. Metab..

[255] Langdahl B.L., Libanati C., Crittenden D.B., Bolognese M.A., Brown J.P., Daizadeh N.S., Dokoupilova E., Engelke K., Finkelstein J.S., Genant H.K. (2017). Romosozumab (sclerostin monoclonal antibody) versus teriparatide in postmenopausal women with osteoporosis transitioning from oral bisphosphonate therapy: A randomised, open-label, phase 3 trial. Lancet.

[256] Cosman F., Crittenden D.B., Adachi J.D., Binkley N., Czerwinski E., Ferrari S., Hofbauer L.C., Lau E., Lewiecki E.M., Miyauchi A. (2016). Romosozumab Treatment in Postmenopausal Women with Osteoporosis. N. Engl. J. Med..

[257] Morales-Santana S., Garcia-Fontana B., Garcia-Martin A., Rozas-Moreno P., Garcia-Salcedo J.A., Reyes-Garcia R., Munoz-Torres M. (2013). Atherosclerotic Disease in Type 2 Diabetes Is Associated With an Increase in Sclerostin Levels. Diabetes Care.

[258] Hampson G., Edwards S., Conroy S., Blake G.M., Fogelman I., Frost M.L. (2013). The relationship between inhibitors of the Wnt signalling pathway (Dickkopf-1(DKK1) and sclerostin), bone mineral density, vascular calcification and arterial stiffness in post-menopausal women. Bone.

[259] Claes K.J., Viaene L., Heye S., Meijers B., d’Haese P., Evenepoel P. (2013). Sclerostin: Another Vascular Calcification Inhibitor?. J. Clin. Endocrinol. Metab..

[260] Cooke M.M., McCarthy G.M., Sallis J.D., Morgan M.P. (2003). Phosphocitrate Inhibits Calcium Hydroxyapatite Induced Mitogenesis and Upregulation of Matrix Metalloproteinase-1, Interleukin-1β and Cyclooxygenase-2 mRNA in Human Breast Cancer Cell Lines. Breast Cancer Res. Treat..

[261] Lin C., Liao W., Jian Y., Peng Y., Zhang X., Ye L., Cui Y., Wang B., Wu X., Xiong Z. (2017). CGI-99 promotes breast cancer metastasis via autocrine interleukin-6 signaling. Oncogene.

[262] Burmester G.R., Lin Y., Patel R., van Adelsberg J., Mangan E.K., Graham N.M.H., van Hoogstraten H., Bauer D., Ignacio Vargas J., Lee E.B. (2017). Efficacy and safety of sarilumab monotherapy versus adalimumab monotherapy for the treatment of patients with active rheumatoid arthritis (MONARCH): A randomised, double-blind, parallel-group phase III trial. Ann. Rheum. Dis..

[263] Huizinga T.W.J., Fleischmann R.M., Jasson M., Radin A.R., van Adelsberg J., Fiore S., Huang X., Yancopoulos G.D., Stahl N., Genovese M.C. (2014). Sarilumab, a fully human monoclonal antibody against IL-6Rα in patients with rheumatoid arthritis and an inadequate response to methotrexate: Efficacy and safety results from the randomised SARIL-RA-MOBILITY Part A trial. Ann. Rheum. Dis..

[264] Emery P., Rondon J., Parrino J., Lin Y., Pena-Rossi C., van Hoogstraten H., Graham N.M.H., Liu N., Paccaly A., Wu R. (2019). Safety and tolerability of subcutaneous sarilumab and intravenous tocilizumab in patients with rheumatoid arthritis. Rheumatology.

[265] Mitoma H., Horiuchi T., Tsukamoto H., Ueda N. (2018). Molecular mechanisms of action of anti-TNF-α agents—Comparison among therapeutic TNF-α antagonists. Cytokine.

[266] Madhusudan S. (2004). A Phase II Study of Etanercept (Enbrel), a Tumor Necrosis Factor Inhibitor in Patients with Metastatic Breast Cancer. Clin. Cancer Res..

[267] Raaschou P., Frisell T., Askling J. (2015). TNF inhibitor therapy and risk of breast cancer recurrence in patients with rheumatoid arthritis: A nationwide cohort study. Ann. Rheum. Dis..

[268] Mamtani R., Clark A.S., Scott F.I., Brensinger C.M., Boursi B., Chen L., Xie F., Yun H., Osterman M.T., Curtis J.R. (2016). Association Between Breast Cancer Recurrence and Immunosuppression in Rheumatoid Arthritis and Inflammatory Bowel Disease: A Cohort Study: BREAST CANCER RECURRENCE WITH IMMUNOSUPPRESSION IN RA AND IBD. Arthritis Rheumatol..

[269] Chiesa Fuxench Z.C., Shin D.B., Ogdie Beatty A., Gelfand J.M. (2016). The Risk of Cancer in Patients With Psoriasis: A Population-Based Cohort Study in the Health Improvement Network. JAMA Derm..

[270] Chen Y., Sun J., Yang Y., Huang Y., Liu G. (2016). Malignancy risk of anti-tumor necrosis factor alpha blockers: An overview of systematic reviews and meta-analyses. Clin. Rheumatol..

[271] Chen Y., Friedman M., Liu G., Deodhar A., Chu C.-Q. (2018). Do tumor necrosis factor inhibitors increase cancer risk in patients with chronic immune-mediated inflammatory disorders?. Cytokine.

[272] Liu Y., Fan W., Chen H., Yu M.-X. (2014). Risk of Breast Cancer and Total Malignancies in Rheumatoid Arthritis Patients Undergoing TNF-α Antagonist Therapy: A Meta-analysis of Randomized Control Trials. Asian Pac. J. Cancer Prev..

[273] Karnoub A.E., Dash A.B., Vo A.P., Sullivan A., Brooks M.W., Bell G.W., Richardson A.L., Polyak K., Tubo R., Weinberg R.A. (2007). Mesenchymal stem cells within tumour stroma promote breast cancer metastasis. Nature.

[274] Cui X., Liu J., Bai L., Tian J., Zhu J. (2014). Interleukin-6 induces malignant transformation of rat mesenchymal stem cells in association with enhanced signaling of signal transducer and activator of transcription 3. Cancer Sci..

[275] Biswas S., Mandal G., Roy Chowdhury S., Purohit S., Payne K.K., Anadon C., Gupta A., Swanson P., Yu X., Conejo-Garcia J.R. (2019). Exosomes Produced by Mesenchymal Stem Cells Drive Differentiation of Myeloid Cells into Immunosuppressive M2-Polarized Macrophages in Breast Cancer. J. Immunol..

[276] Ijiri K., Nagayoshi R., Matsushita N., Tsuruga H., Taniguchi N., Gushi A., Sakakima H., Komiya S., Matsuyama T. (2002). Differential Expression Patterns of Secreted Frizzled Related Protein Genes in Synovial Cells from Patients with Arthritis. J. Rheumatol..

[277] Imai K., Morikawa M., D’Armiento J., Matsumoto H., Komiya K., Okada Y. (2006). Differential expression of WNTs and FRPs in the synovium of rheumatoid arthritis and osteoarthritis. Biochem. Biophys. Res. Commun..

